# Structural and functional characteristics and expression profile of the 20S proteasome gene family in Sorghum under abiotic stress

**DOI:** 10.3389/fpls.2023.1287950

**Published:** 2023-11-29

**Authors:** Vijai Malik, Faiza Mohamad Ikram, Yogita Singh, Vivek Kumar, Pranita Malik, Priyanka Balyan, Krishna Pal Singh, Reyazul Rouf Mir, Abeer Hashem, Graciela Dolores Avila-Quezada, Elsayed Fathi Abd_Allah, Upendra Kumar

**Affiliations:** ^1^ Department of Botany, Chaudhary Charan Singh University, Meerut, India; ^2^ Department of Molecular Biology & Biotechnology, College of Biotechnology, Chaudhary Charan Singh (CCS) Haryana Agricultural University, Hisar, India; ^3^ Department of Botany, Deva Nagri P.G. College, Chaudhary Charan Singh (CCS) University, Meerut, India; ^4^ Biophysics Unit, College of Basic Sciences & Humanities, Govind Ballabh (GB) Pant University of Agriculture & Technology, Pantnagar, India; ^5^ Mahatma Jyotiba Phule Rohilkhand University, Bareilly, India; ^6^ Division of Genetics and Plant Breeding, Sher-e-Kashmir University of Agricultural Sciences and Technology of Kashmir (SKUAST-Kashmir), Srinagar, India; ^7^ Botany and Microbiology Department, College of Science, King Saud University, Riyadh, Saudi Arabia; ^8^ Facultad de Ciencias Agrotecnológicas, Universidad Autónoma de Chihuahua, Chihuahua, Chihuahua, Mexico; ^9^ Plant Production Department, College of Food and Agricultural Sciences, King Saud University, Riyadh, Saudi Arabia; ^10^ Department of Plant Science, Mahatma Jyotiba Phule Rohilkhand University, Bareilly, India

**Keywords:** *Sorghum bicolor*, 20S proteasome gene family, gene duplication, miRNAs, splice variants, homology modelling, cis-regulatory elements

## Abstract

The 26S proteasome is a molecular machine that catalyzes and degrades protein intracellularly with the help of its core complex called 20S proteasome. The 20S proteasomes degrade and cleave denatured, cytotoxic, damaged, and unwanted proteins via proteolysis and impart biotic and abiotic stress tolerance in model plants. This study identified 20 genes, namely, 10 *SbPA* and 10 *SbPB* that encode for α- and β-subunits of the 20S proteasome in *Sorghum bicolor* (L.) Moench (2n= 20). These genes have been found distributed on the 1st, 2nd, 3rd, 4th, 5th, 7th, and 10th chromosomes. These sorghum genes were orthologous to corresponding rice. Phylogenetic analysis clustered these genes into seven clades, each with one of the seven α-subunits (1 to 7) and one of the seven β-subunits (1 to 7). *In silico* gene expression analysis suggested that nine genes were involved in abiotic stress response (cold, drought, and abscisic acid hormone). The expression of these proteasomal genes was studied in shoots and roots exposed to different abiotic stresses (cold, drought, and abscisic acid) by quantitative real-time polymerase chain reaction. A significant increase in the relative fold expression of *SbPBA1*, *SbPAA1*, *SbPBG1*, *SbPBE1*, and *SbPAG1* genes under ABA and drought stress provides an insight into its involvement in abiotic stress. No expression was observed for cold stress of these genes indicating their non-involvement. It is believed that additional investigation into the *SbPA*/*SbPB* genes would aid in the creation of *S. bicolor* cultivars that are resistant to climate change.

## 1 Introduction

*Sorghum bicolor*, a coarse grain, is primarily used as food and fodder in Asia, Africa, the Americas, and Australia. Besides fodder, like sugarcane, the juicy stalks of sweet *S. bicolor* can be utilized for preparation of syrup and jaggery. Grain from *S*. *bicolor* is germinated, dried, and processed to create malt, which serves as a substratum for fermentation in the creation of beer. India ranks second in the world for the cultivation (6.18 million hectares) and production (5.28 million tons) of *S. bicolor*. It is the third cereal crop after rice and wheat. For several decades, the main focus of research has been on developing agricultural cultivars that can survive biotic and abiotic stresses (Dhankher and Foyer, 2018; Kumar et al., 2019). Abiotic stress mainly involves temperature, salinity, and drought, causing crop yield losses, whereas biotic stresses include different fungal, bacterial, and viral diseases and insect pests (Sun et al., 2019). In response to these stresses, the physiological and molecular responses induce the proteolytic capacity of the eukaryotic cells to selectively remove/degrade the unnecessary/damaged proteins (Kurepa et al., 2009).

The ubiquitin–proteasome system (UPS) is an important protein degradation pathway that removes nuclear, cytosolic, and other membrane proteins (Glickman and Ciechanover, 2002; Finley, 2009). The 26S proteasome holoenzyme of UPS consists of a 19S regulatory particle and a 20S core particle, which recognizes ubiquitin signals and hydrolyzes unfolded polypeptides into short peptides (Saeki, 2017; Yu and Matouschek, 2017). The core complex is integral to the proteasome and is found in many cell types. Under stress, the 20S particle removes misfolded or damaged proteins, catalyzing protein degradation in a non-lysosomal, ATP-dependent manner. The 20S proteasome, a barrel shaped structure, forms the core of the 26S. 20S is made up of 28 subunits arranged in four rings of 7 α- and 7 β-subunits placed one above the other. In the center, two identical rings are made up of 7 β-subunits each. Terminal rings are made up of 7 α-subunits. This gives a symmetric (α 1-7/β 1-7/β 1-7/α 1-7) organization to 20S proteasome. Three β-subunits (numbered β1, β2, and β5) in the middle rings have a proteolytic active site with a specific substrate preference. The α-subunits at the terminal end of 20S proteasome are responsible for entry and exit of peptides. In *Arabidopsis thaliana* and many eukaryotes, there are 7 α-subunits and 7 β-subunits. Nomenclaturally, these α- and β-subunits can be represented as PAA-PAG and PBA-PBG, respectively.

Genetics of UPS has been figured out in species like Arabidopsis, rice (Fu et al., 1998), wheat (Sharma et al., 2022), and rapeseed (Kumar et al., 2022). In wheat, the 20S proteasome genes were identified and characterized, providing their roles in different biological processes related to abiotic stress tolerance (Sharma et al., 2022). Additionally, it has been demonstrated that the genes encoding various 20S proteasome subunits in rapeseed are linked to biotic and abiotic stressors (Kumar et al., 2022). Several other functions such as tolerance to arsenic (Sung et al., 2016), antiviral response (Dielen et al., 2011), immune response, and organellar stress inducing programmed cell death (L. Sun et al., 2013; Sun H.H et al., 2013; Cai et al., 2018) are also contributed by the 20S proteasomal genes. In Arabidopsis, *AtPBE1* regulates proteasome assembly under salt stress (Han et al., 2019). In maize, *AtPBAC4* ortholog resulted in defective/collapsed kernels (Wang et al., 2019). In wheat, *TaGW2*, which encodes the E3 ring ligase of proteasomal complex, regulates the grain size (Song et al., 2007). In rice, *OgTT1* (α2 subunit) is associated with heat tolerance and their adaptation (Li et al., 2015).

Data mining computational/bioinformatic approaches provide us an opportunity to access the information available in the databases and have augmented to the structure of system biology (Swarbreck et al., 2007). A number of accessible computational tools/methods, databases, and experimental datasets of many crops are helpful in providing understanding of gene structure, duplication events, molecular phylogeny, cis regulatory elements distribution, microRNA target prediction, differential expression, gene interactions, coexpression networks, gene ontology (GO), and post-transcriptional/translational modifications (Toufighi et al., 2005; Kurata and Yamazaki, 2006; Swarbreck et al., 2007; Hruz et al., 2008; Lyons and Freeling, 2008; Bailey et al., 2009; Letunic et al., 2009; Proost et al., 2009; Sakai et al., 2013; Hu et al., 2015; Chow et al., 2016; Letunic and Bork, 2016; Xia et al., 2017; Dai et al., 2018; Ge et al., 2020).

To date, the comprehensive investigations on structural and functional aspects, and expression profiles of 20S proteasomal genes in sorghum have not been undertaken. Here, we aimed to identify the 20S proteasomal candidate genes, analyzed their structural features, and investigated promoter structure prediction and cis regulatory elements, transcriptional and post-transcriptional regulation, and also their *in silico* expression profiles at different stages of development and under different abiotic stresses, to understand the roles of these genes in the degradation pathway. Understanding the genetic architecture of 20S proteasomal genes involved in plant’s stress physiology and development ultimately provides a new foundation for future research into development of climate-resilient/smart *S. bicolor*.

## 2 Materials and methods

### 2.1 Characterization of 20S proteasome family genes and proteins

#### 2.1.1 Identification and sequence retrieval of candidate genes

The sequences of 20S proteasome genes for *S. bicolor* and rice are available in the ensembl database (https://plants.ensembl.org/index.html). The coding sequences (CDS) of 23 rice genes (OsPA/OsPB) available in the ensembl database (https://plants.ensembl.org/index.html) have been utilized to obtain the 20S proteasome gene sequences of *S. bicolor*. The gene sequences of *S. bicolor* have been searched using Tblastx (e value ≤ 1e^-^5) against the available rice genome assembly. The hits were examined for the presence of specific domains as available in query sequences using the conserved domain database (CDD) (Lu et al., 2020) search tool at NCBI. High-level query coverage and (more than 60%) sequence similarity, and the presence of all domains and motifs available in query sequences were the criteria that were used to identify the orthologs in Sorghum.

#### 2.1.2 Gene structure, duplication and genome-wide localization

The structure of gene was analyzed using ensembl plants (https://plants.ensembl.org/index.html), the gene structure display server (http://gsds.gao-lab.org) (Hu et al., 2015), meme suits (https://meme-suite.org/meme/tools/meme) (Bailey et al., 2009), and TB tools (https://github.com/CJ-Chen/TBtools/releases) (Chen et al., 2020). Repeat masker Version: open-4.0.9 (https://www.repeatmasker.org/cgi-bin/WEBRepeatMasker) (Chen, 2004) has been utilized to identify SSRs in gene sequences. All these were done using default and modified parameters.

#### 2.1.3 Promoter structure analysis and miRNA target prediction

Utilizing Plant CARE (http://bioinformatics.psb.ugent.be/webtools/plantcare/html/) (Lescot et al., 2002), it was possible to find cis regulatory elements in the gene sequence upstream of 1,500 bp from the promoter region. The probable micro-RNAs and their targets in the genes of *S. bicolor* were looked for using the default parameters of the web-based service psRNATarget (https://www.zhaolab.org/psRNATarget/) (Dai et al., 2018). Here, e-value 0–3 was applied (Rensink & Buell, 2004; Lu et al., 2019).

#### 2.1.4 Molecular phylogeny, synteny, and collinearity

Ensembl plants gene tree pipeline (protein sequence alignments) (Rogozin et al., 2003; Yu et al., 2019) was utilized to establish evolutionary relationship among proteasome genes using gene identifier for each gene of *S. bicolor*. Using the plant compara option, a gene tree of homologs across the genomes of *S. bicolor* and rice was created. This gene tree can be used to identify duplication and speciation events called paralogy and orthology, respectively. Ka/Ks ratios for seven pairs of gene were calculated using tbtool. Synteny/collinearity of *S. bicolor* with rice gene was determined using blocks of 25 genes. Genomicus tool v. 49.01 (https://www.genomicus.bio.ens.psl.eu/genomicus-plants-49.01/cgi-bin/search.pl) (Muffato et al., 2010) was used for this purpose.

#### 2.1.5 Physio-chemical properties of candidate proteins

The NCBI keeps a database of conserved domains that can be searched on CDs (CDD). The major domains in the protein sequences of *S. bicolor* were identified using this tool. Rice has been manually searched for α/β-proteasome domains, which are distinctive traits of the proteasome family. Physio-chemical parameters such as amino acid composition, molecular weight, theoretical PI, the number of positively and negatively charged residues, instability index, aliphatic index, GRAVY, and stability have been calculated using the Protparam tool (https://web.expasy.org/protparam/) (Laskowski et al., 1993) of Expasy. Similar to this, the SOPMA tool (Geourjon and Deleage, 1995) was used to calculate the secondary level characteristics of proteins (https://npsa-prabi.ibcp.fr/cgi-bin/npsaautomat.pl?page=/NPSA/npsasopma.html).

#### 2.1.6 Conserved motifs discovery and homology modeling

Meme suite (https://meme-suite.org/meme/tools/meme) was utilized to search motifs. Inter proscan database (https://www.ebi.ac.uk/interpro/search/sequence/) was utilized to annotate identified motifs. Homology modeling was used to infer the predicted proteins’ 3D structures. Pdb was tested using the Swiss model template library (https://swissmodel.expasy.org/). Geometric and energetic validation of the predicted 3D protein structures was done using the structural analysis and validation system (https://saves.mbi.ucla.edu/). Saves v6.0’s option procheck was used to compare the relative proportion of amino acids in a favored region to another region (Li et al., 2015). The quality of the protein was checked through the PROCHECK server by dihedral analysis of the Ramachandran plots of predicted candidate proteins. VERIFY-3D (Eisenberg et al., 1997) was used to evaluate how well the atomic model (3D) matched the amino acid sequence (Clavijo et al., 2017). To examine the statistics of non-bonded interactions between various atom types, the Errat option of saves was employed (Parmentier et al., 1997).

#### 2.1.7 Structural characterization, subcellular localization, and gene ontology analysis

By aligning their representative structures, the 3D structures of proteins predicted for *S*. *bicolor* and those encoded by various *Oryza sativa* genes were compared using the Fat Cat server (https://fatcat.godziklab.org/fatcat/fatcat pair.html). By comparing the root mean square deviation (RMSD) values of the C_α_ atoms of the created structures to those of the corresponding 3D structures of the query genes, the similarity of the generated 3D structures in a globally optimized superimposition environment was determined. Subcellular localization and GO analysis were conducted using the Biomart tool (https://plants.ensembl.org/biomart/martview/1e86aac7e869419fd945a124d55c0405) (Smedley et al., 2009) and ShinyGO tool (http://bioinformatics.sdstate.edu/go/) (Ge et al., 2020), respectively. The protein–protein interaction network was performed to uncover unknown functions of proteins at the molecular level using string database (Szklarczyk et al., 2023).

#### 2.1.8 Sequence alignment and phylogenetic analysis

To identify conserved and coevolving amino acid residues in *S*. *bicolor* and rice, multiple sequence alignment was performed using the MultAlin tool (http://multalin.toulouse.inra.fr/multalin/). The mutual information (MI) between two amino acid locations in MSA was calculated using the Mistic web server (http://mistic.leloir.org.ar/results.php?jobid=202112021211022296). Coevolving residues were found using MI. The MI between two places (two columns in the MSA) measures how well we can anticipate the amino acid identification at a different position when we are aware of an amino acid at one position. Thus, MI is a measurement that enables the detection of associated and compensating mutation sites in homologous proteins. Using MEGA software version 6.0 (MEGA stands for molecular evolutionary genetics analysis), the phylogenetic analysis of protein amino acid sequences was carried out (Xu & Xue, 2019). To create an unrooted tree, a neighbor-joining approach with a bootstrap requiring 1,000 iterations was used. Mega software’s Newick format tree was developed with iTOL (https://itol.embl.de/) (Letunic and Bork, 2016).

### 2.2 *In silico* expression profiling

The expression profiling of all the candidate *SbPA*/*SbPB* genes at different levels was performed using GENEVESTIGATOR (https://genevestigator.com/) (Hruz et al., 2008). First, at the tissue-specific level, the expression was studied on a quantitative basis in different tissues of sorghum, taking into account the SB-mRNASeq-SORGHUM datasets and selecting all the 20 *SbPA*/*SbPB* genes in a heat map format. Second, a set of experiments were performed for the 20 *SbPA*/*SbPB* genes at 10 different developmental stages. Third, the expression of these 20 *SbPA*/*SbPB* genes was studied under abiotic (cold, drought, and ABA hormone) stress and the data were presented in the form of fold values.

### 2.3 Validation of proteasomal genes through qRT-PCR

For experimental validation of candidate genes, the sorghum cultivar HJ 541 was selected and their seeds were grown in soil-filled pots under greenhouse conditions. Various abiotic stress treatments (cold: 4°C, ABA and drought: 10% of soil moisture remaining in pot) were performed. The leaf and root samples were collected from 4-week-old plants after providing the abiotic stresses for 10 days. Total RNA was isolated from samples using the Maxwell RSC Plant RNA kit (Promega, United States), according to manufacturer’s protocol. From the isolated RNA, cDNA was synthesized using the RevertAid cDNA synthesis kit (Thermo Scientific, United States). The Primer Quest tool of IDT was used to design specific primers for quantitative expression analysis. *Actin* was used as an endogenous control. Quantitative real-time polymerase chain reaction was performed using Quant Studio 6 Flex system, with three biological replicates. Relative expression of genes was quantified using the 2^−ΔΔCT^ method to identify the expression pattern of proteasomal genes under abiotic stress.

## 3 Results

**Figure 1:**
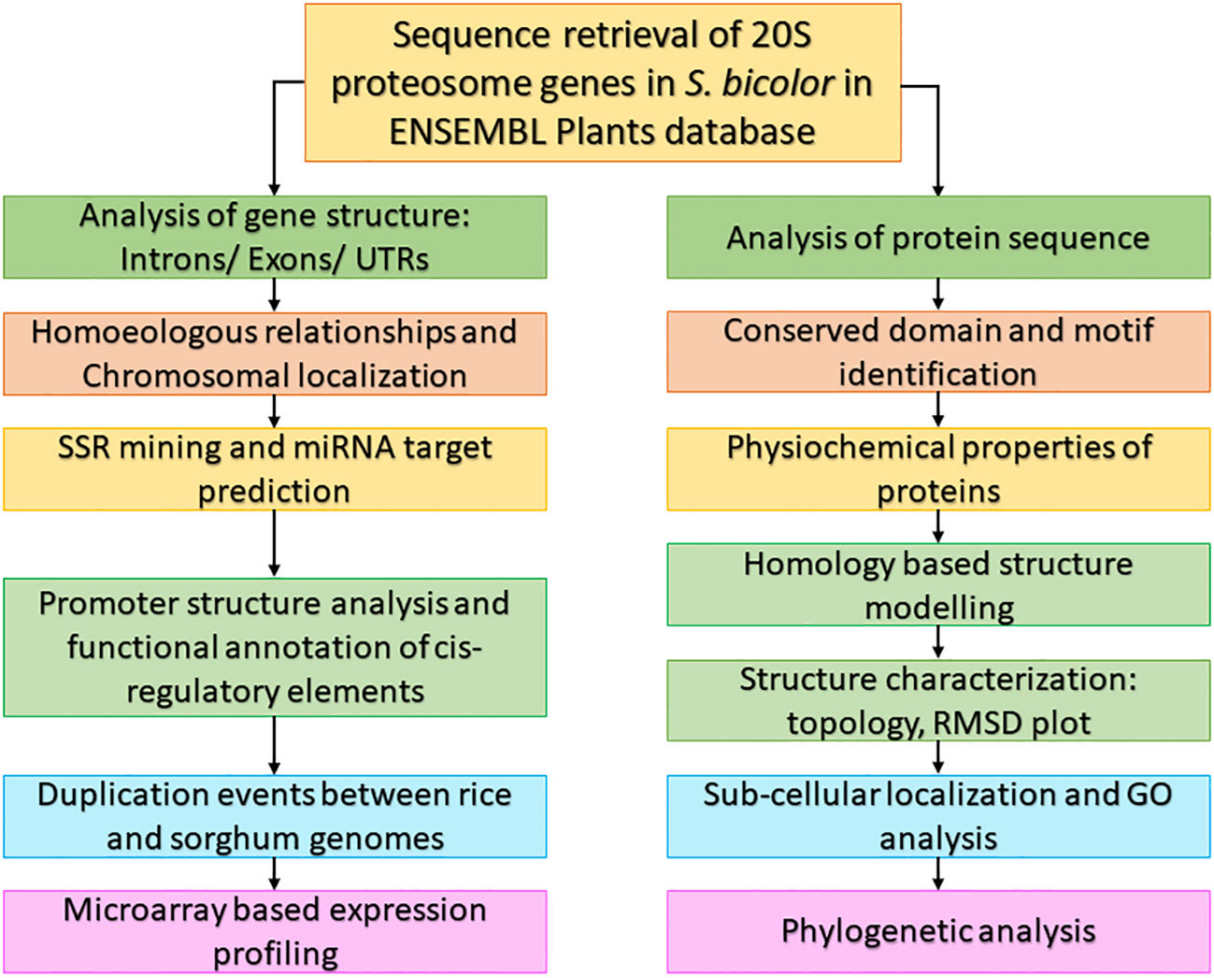
Workflow used to characterize 20S proteasomal genes in *S. bicolor*.

Numerous crops’ sequenced plant genomes have been used to research genes involved in various developmental stages and stress tolerance (Lu et al., 2019). The sequenced genomes of model plants *A. thaliana* and *O. sativa*, which serve as a platform for comparative studies, are beneficial to crops whose genomes have not been sequenced (Rensink and Buell, 2004). The workflow used to characterize the 20S proteasomal genes in this study is given in **Figure 1**.

### 3.1 Gene sequence analysis

#### 3.1.1 Gene structure, splice variants, and chromosomal location of 20S proteasome genes

The present investigation revealed 20 proteasomal genes in *S. bicolor*. These genes were classified into seven distinct α and β types of the 20S proteasome family. **Table 1** contains detailed information about these genes, as well as the cDNA and CDS sequences of the α-subunits (SbPAA-SbPAG) and β-subunits (SbPBA-SbPBG). Information on homology of *S. bicolor* with rice is given in **Table 1**. All the 20 Sorghum genes have been designated on the basis of corresponding genes reported in rice (Fu et al., 1998; Livneh et al., 2016).

**Table 1:** Detailed information about genes, cDNA, and CDS sequences for α- and β-subunits of 20S proteasome in *S*. *bicolor* and rice.

Sorghum bicolor				Oryza sativa			
Gene name	Gene length bp	cDNA length bp	CDS length bp	Gene name	Gene length bp	cDNA length bp	CDS length bp
α- subunit							
SbPAA1	2,701	1,026	741	OsPAA1	4,204	1,047	741
				OsPAA2	4,485	1,609	762
SbPAB1	5,975	1,205	708	OsPAB1	4,412	1,133	708
SbPAB2	4,416	1,187	708	OsPAB2	4,204	995	708
SbPAC1	2,221	2,221	753	OsPAC1	1,077	1,077	753
				OsPAC2	1,077	1,077	753
				OsPAC3	956	956	753
SbPAD1	2,289	1,293	750	OsPAD1	2,195	949	750
				OSPAD2	3,396	1,040	750
SbPAE1	2,701	1,132	714	OsPAE1	4,304	1,187	714
SbPAE2	4,955	1,165	714				
SbPAF1	3,660	1,199	813	OsPAF1	3,416	1,078	813
SbPAG1	9,357	612	612	OsPAG1	4,419	1,188	750
SbPAG2	5,860	1,655	750	OsPAG2	4,148	1,141	750
β-subunit							
SbPBA1	6,010	3,709	738	OsPBA1	3,530	1,065	741
SbPBA2	3,737	1,152	738	OsPBA2	3,357	999	741
SbPBB1	4,655	1,451	822	OsPBB1	3,111	1,194	819
SbPBB2	3,408	1,344	822				
SbPBC1	3,913	1,581	615	OsPBC1	3,374	1,038	615
SbPBC2	3,651	1,580	615	OsPBC2	2,500	877	615
SbPBD1	2,789	1,202	633	OsPBD1	2,346	968	639
SbPBE1	2,873	1,321	843	OsPBE1	2,943	1,217	834
SbPBF1	2,657	1,064	660	OsPBF1	3,440	1,039	666
				OsPBF2	2,186	1,063	660
SbPBG1	2,401	1,121	792	OsPBG1	2,517	1,090	771

Sb, Sorghum bicolor; Os, Oryza sativa; CDS, coding sequence; cDNA, complementary DNA; Bp, base pair; PA, proteasome alpha (α); PB, proteasome beta (β).

**Figure 2:**
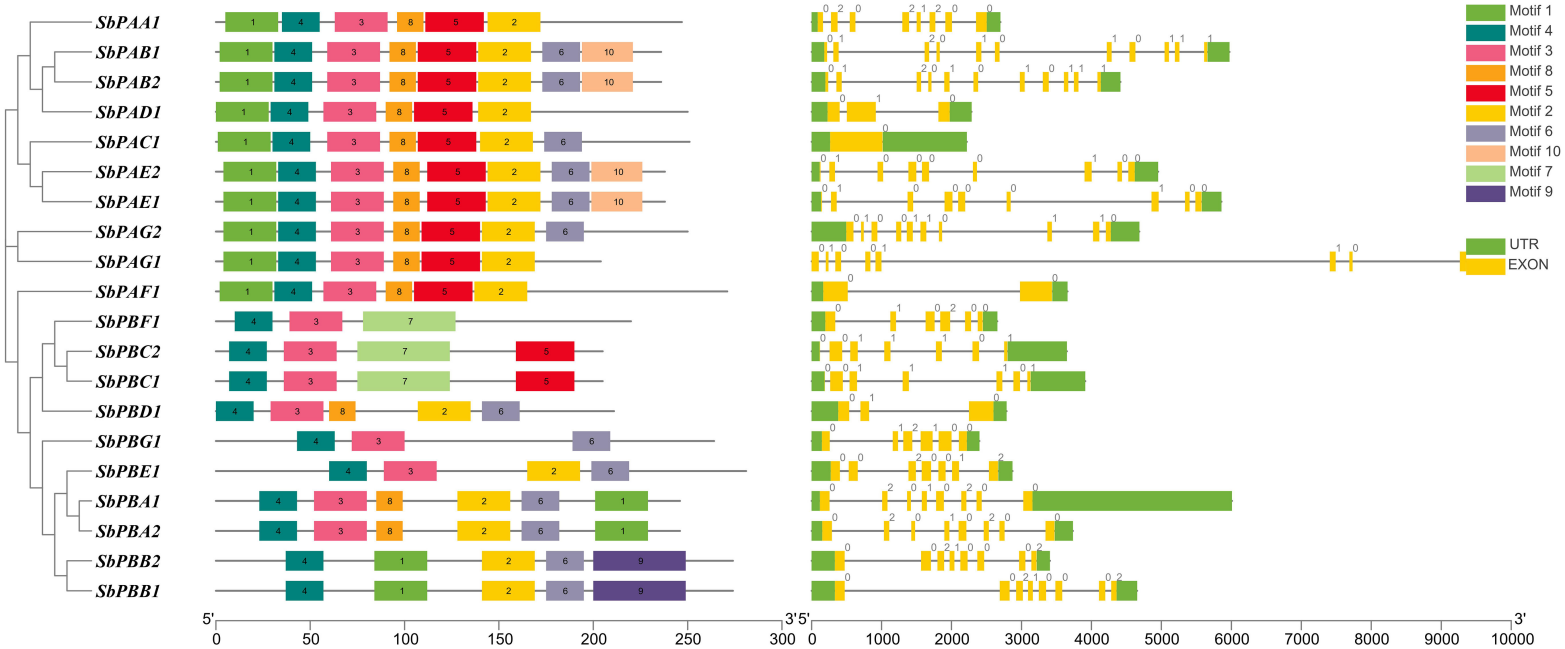
Structure of SbPA and SbPB genes of *Sorghum bicolor* showing distribution of exons (yellow solid bars), introns (black lines), upstream/downstream regions (solid green bars), and intron phases marked as 0, 1 and 2. This figure also represents the conserved motifs identified in SbPA and SbPB proteins.

**Figure 3:**
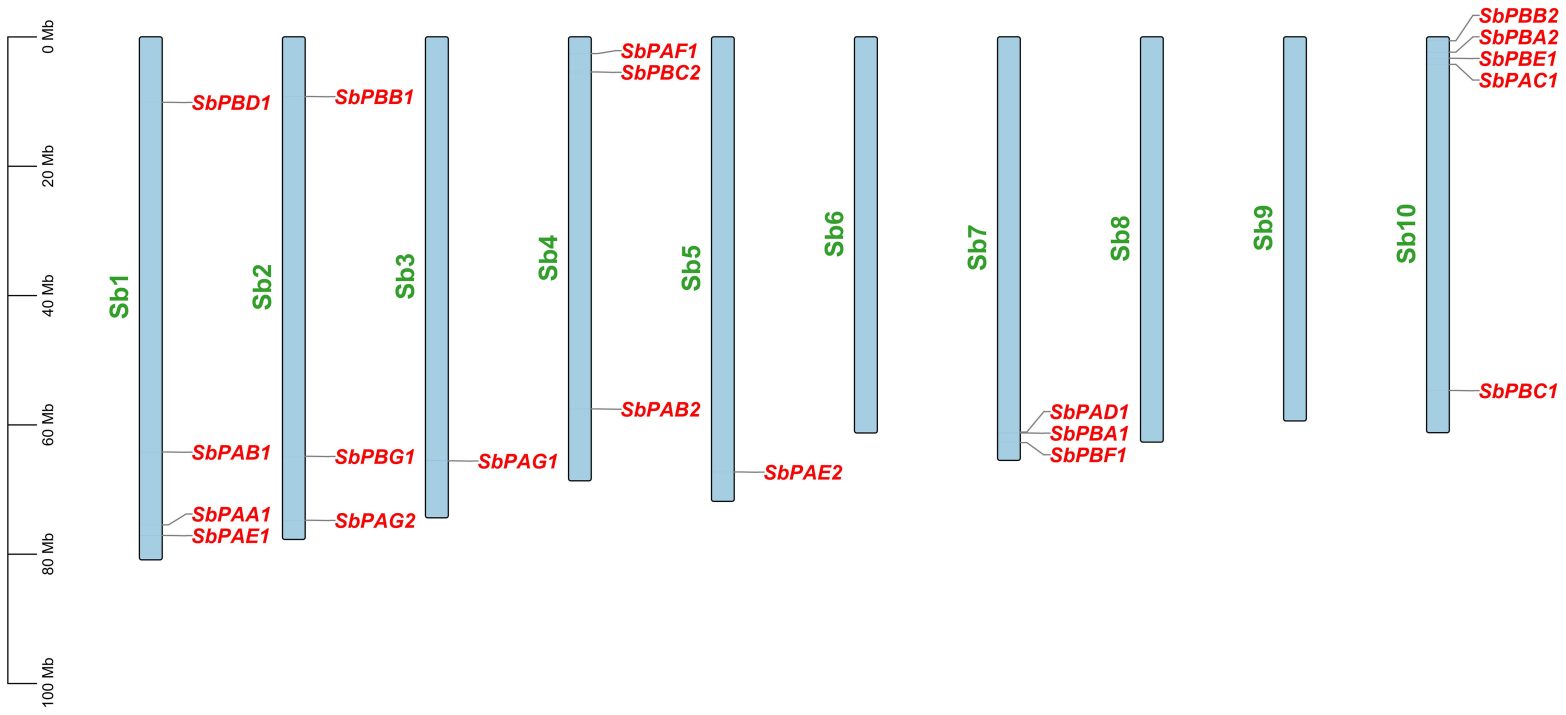
Distribution of 20 *SbPA* and *SbPB* genes on 20 chromosomes of *Sorghum bicolor* belonging to 1–10 chromosomes. On each chromosome, gene names are given on the upper side and their physical positions in megabases (Mb) are indicated on the left.

Each *SbPA* gene has a length that varied from 2,221 to 9,357 base pairs. Exon and intron count in *SbPA* genes ranged from 1 to 11, respectively. The 10 *SbPA* genes are all intron-containing The *SbPB* genes’ lengths ranged from 2,401 to 6,010 base pairs. Exon and intron count in SbPB genes ranged from 3 to 8 and from 2 to 7, respectively (**Supplementary Table 1**). Although there are some exceptions, the structural arrangement of exon and intron was found to be comparable in the majority of *SbPA* and *SbPB* genes. The *SbPA* gene’s cDNA sequence ranged from 612 to 2,221, while the *SbPB* gene’s sequence ranged from 1,064 to 3,709 (**Supplementary Table 1**). *SbPA* (708–813) and *SbPB* (615–843) genes showed individual variations in CDS. **Figure 2** shows the distribution of exons (solid yellow bar), introns (black lines), upstream and downstream areas (solid blue bar), and UTR (3’ or 5’) (solid green bar), as well as intron phase 0 (56.83%), phase 1 (30.93%), and phase 2 (12.23%). *SbPA* and *SbPB* genes were found to be unevenly distributed throughout 10 chromosomes, which may be the result of gene duplication or gene loss (Li et al., 2015; Clavijo et al., 2017; Yu et al., 2019). The Chromosome Sb10 had the maximum number (5) of genes (*SbPAC1*, *SbPBA2*, *SbPBB2*, *SbPBC1*, and *SbPBE1*) and chromosomes Sb3 and Sb5 had one gene each. Chromosomes Sb6, Sb8, and Sb9 are without any gene. The positions of all the genes were terminal and sub-terminal (**Figure 3**). In contrast to the prior work by Sassa et al., which reported that there were 14 genes in rice, the phylogenomic survey found that there are 23 genes (Sassa et al., 2000). More thorough phylogenomic surveys may also be able to resolve duplications and heterogeneity in other organisms.

Using gene trees that included Sorghum genes as well as genes from *O. sativa*, the orthology between 10 *SbPA* and 10 *SbPB* genes was studied. Utilizing the Ensembl Plant Compara pipeline, this tree was created (**Supplementary Figure 1**).

#### 3.1.2 Gene duplication and synteny

The orthologous and paralogous interactions between the *SbPA* and *SbPB* genes in several taxa are outlined in **Supplementary Table 2**. The 23 genes (13 α and 10 β) in rice were divided into seven duplicates, one triple, and the remaining six singletons (**Supplementary Table 2**). The duplication pattern in rice was used to predict the comparable pattern in *S. bicolor*. Using the plant compara gene tree in the ensembl plant database allowed us to distinguish orthologs from paralogs. Seven gene pairs have been found in the peptides of *Sorghum bicolour* and rice. Ka and Ks ratios for all the seven pairs were found to be less than one. These values indicate stabilizing selection, i.e., genes are constrained to maintain their current function and thus acting against change favoring conservation (**Supplementary Table 3**). Synteny of 20 genes of *S. bicolor* with the rice gene has been found to be 100% (**Supplementary Figure 2**). However, the collinearity of genes of *S. bicolor* with rice was absent.

#### 3.1.3 SSRs mining

Eleven (55%) of the 20 genes included a total of 22 SSRs. This suggests that only a small fraction of the genes in a gene family include SSRs. Of these, 14 SSRs were found in eight *SbPA* genes and eight SSRs in three *SbPB* genes. The number of SSR per gene also varied, with two genes (*SbPAG2* and *SbPBB1*) having five SSRs, three genes (*SbPAE2*, *SbPAG1*, and *SbPBB2*) having two SSRs, and the remaining six genes having one SSR each (**Supplementary Table 4**). The most common SSRs (5) have hexanucleotide and trinucleotide motifs, followed by those with mononucleotide (3), dinucleotide (3), pentanucleotide (3), tetranucleotide (2), and heptanucleotide (1) motifs.

#### 3.1.4 Promoter structural analysis and functional annotation of cis-regulatory elements

**Figure 4:**
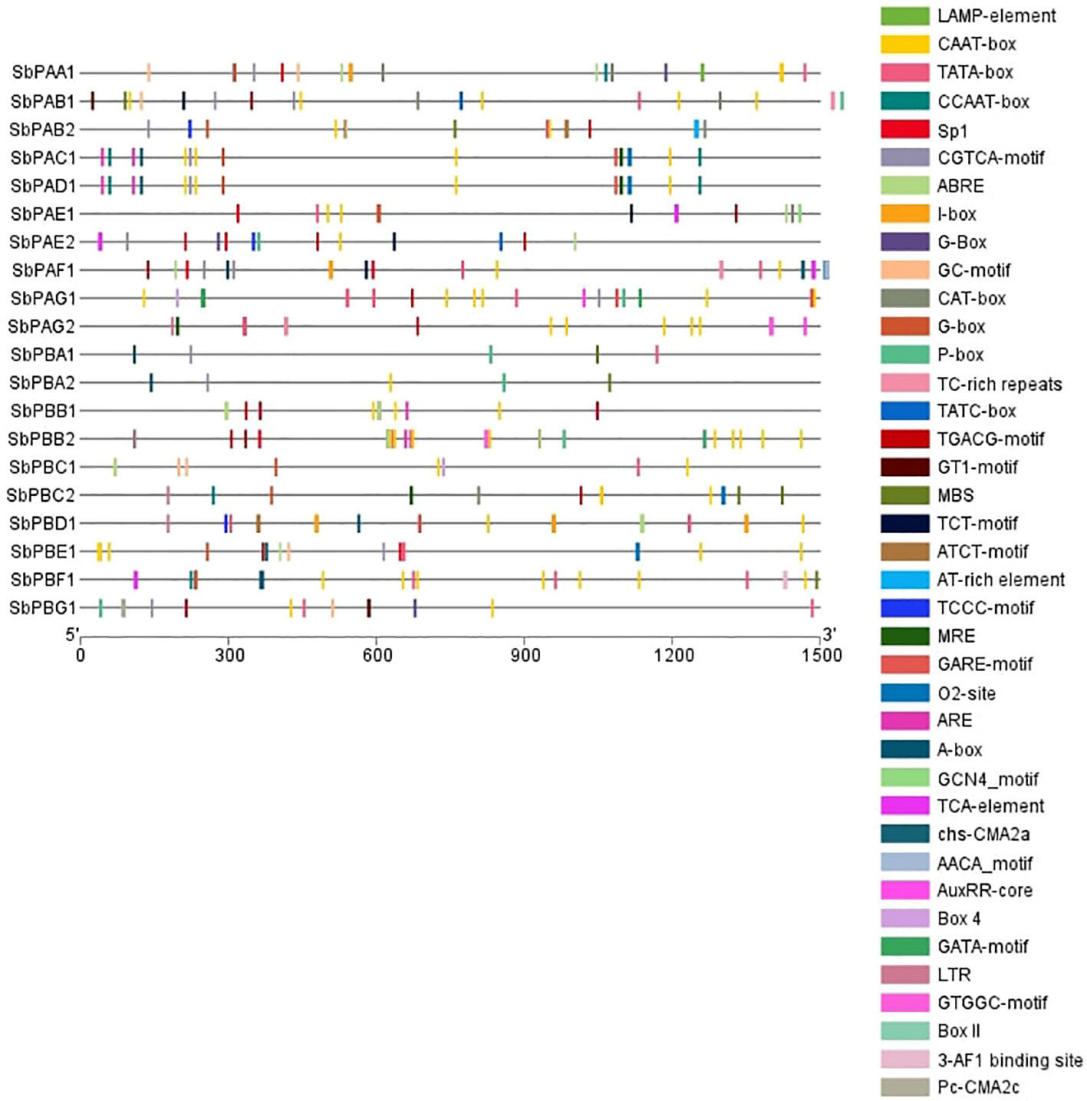
Promoter structure prediction in *S. bicolor.* Different colors depict the presence of identified cis regulatory elements.

All genes’ promoter regions were found to include many LAMP elements, CAAT-box, TATA-box, CCAAT-box, Sp1, CGTCA-motif, ABRE, I-box, G-Box, GC-motif, CAT-box, P-box, TC-rich repeats, TATC-box, TGACG-motif, GT1-motif, MBS, TCT-motif, ATCT-motif, AT-rich element, TCCC-motif, MRE, GARE-motif, O2-site, ARE, A-box, GCN4_motif, TCA-element, chs-CMA2a, AACA motif, AuxRR-core, Box 4, GATA-motif, LTR, GTGGC-motif, Box II, the 3-AF1 binding site, and the Pc-CMA2c elements. Among these, CAAT-box, TATA-box, TGACG-motif, G-Box, MRE, TCT-motif, Sp1, and AT-rich elements were present in maximum number of genes (**Figure 4**). The CAT box and GCN4 motif were found responsive in the expression of meristem and endosperm, respectively (**Supplementary Figure 3**). All of the transcription factors have the A-box motif, which is a cis-acting regulatory element connected to P-box and L-box. It is involved in transcriptional activity that is triggered. In the promoter regions, A-box from various families of transcription factors was discovered.

#### 3.1.5 miRNA target prediction

In the current investigation, we discovered 21 miRNAs that contained the *SbPA* and *SbPB* gene sequences. Only nine genes (six *SbPA* and three *SbPB*) were predicted to include the target locations for these miRNAs. Six miRNAs, the maximum number of target sites, were available for one gene (*SbPBA1*). The targets for *SbPAC1* and *SbPBC2* were available for three miRNAs. Similarly, the targets for *SbPAB2*, *SbPAE1*, and *SbPAG2* were available for two miRNAs, and the targets for *SbPAD1*, *SbPAE2*, and *SbPBC1* were available for one miRNA each. On numerous Sorghum chromosomes, various *SbPA* and *SbPB* genes served as the targets for the remaining 13 miRNAs (**Table 2**).

**Table 2:** Putative miRNAs involved in post-transcriptional regulation of 20S proteasomal genes in *S. bicolor*.

Target genes	miRNA	Length of miRNA	Target position	miRNA sequence	Inhibition
SbPAB2	sbi-miR396d	22	240–261	CUCCACAGGCUUUCUUGAACUG	Cleavage
SbPAB2	sbi-miR396e	22	240–261	UUCCACAGGCUUUCUUGAACUG	Cleavage
SbPAC1	sbi-miR172e	21	1,979–1,999	UGAAUCUUGAUGAUGCUGCAC	Cleavage
SbPAC1	sbi-miR172f	21	1,979–1,999	AGAAUCCUGAUGAUGCUGCAC	Cleavage
SbPAC1	sbi-miR6229-3p	24	392–415	GUUUUUCUCGCCGGGUGAGAAGGC	Cleavage
SbPAD1	sbi-miR5388	22	763–784	AUCUUUGCCGGGUGUCUCUGAC	Cleavage
SbPAE1	sbi-miR6218-3p	21	328–348	ACAAGUUUCGUGAUUUUUGGA	Cleavage
SbPAE1	sbi-miR529	20	53–73	CUGUACCCUCUCUCU-UCUUC	Cleavage
SbPAE2	sbi-miR6223-5p	21	112–132	UUCUUGGGAGGAGCAUGCUAG	Cleavage
SbPAG2	sbi-miR393a	21	1,269–1,290	UCCAAAGGGAUC-GCAUUGAUC	Cleavage
SbPAG2	sbi-miR393b	21	1,269–1,290	UCCAAAGGGAUC-GCAUUGAUC	Cleavage
SbPBA1	sbi-miR6225-5p	24	1,140–1,163	AACUAGACUCAAAAGAUUCAUCUC	Cleavage
SbPBA1	sbi-miR5567	24	1,069–1,092	UUAAUGAUUCAUGUAUGUGUCCAA	Cleavage
SbPBA1	sbi-miR6225-5p	24	1,277–1,300	AACUAGACUCAAAAGAUUCAUCUC	Translation
SbPBA1	sbi-miR5567	24	956–979	UUAAUGAUUCAUGUAUGUGUCCAA	Cleavage
SbPBA1	sbi-miR6228-3p	24	2,545–2,568	GUGGCAGUAGAAUUAAUGAAGGGA	Cleavage
SbPBA1	sbi-miR6228-5p	24	968–991	UUCUAUCUCUAUUAAUUGUGUUGC	Cleavage
SbPBC1	sbi-miR6230-3p	21	811–831	UAACAAGUUUAGGGAUCUAGA	Translation
SbPBC2	sbi-miR5565e	19	1,359–1,377	UUGUUUGGAUGUUGUCGGA	Cleavage
SbPBC2	sbi-miR5386	20	140–158	CGUCGCUGUCGCGCGCGCUG	Cleavage
SbPBC2	sbi-miR6231-3p	21	556–576	UAUUUGUGGACUCAUGGACAU	Cleavage

Most of the miRNAs suppressed the expression of genes with miRNA target sites through post-transcriptional cleavage while the two miRNAs (miR6225-5p and miR6230-3p) suppressed the gene expression through translational inhibition (**Table 2**).

### 3.2 Protein analysis

#### 3.2.1 Physicochemical properties of SbPA and SbPB proteins

Twenty SbPA/SbPB proteins have an average of 242 amino acids (aa), ranging from 204 (SbPAG1, SbPBC1, and SbPBC2) to 280 (SbPBE1). There were identified α-subunits with a length of 204 to 280 aa and β-subunits with a length of 204 to 270 aa (**Supplementary Table 5**). SbPA and SbPB proteins were identified to have a molecular weight range of 22,375.5 kDa (SbPAG1) to 30,218 kDa (SbPBE1). The range of the isoelectric point (PI) was 4.72 to 8.26. While the remaining proteins (16) with greater aliphatic indexes (71.5 to 100.8) were stable, the unstable proteins (4) had aliphatic indexes ranging from 79.22 to 87.26. The protein solubility value was represented by the hydropathy’s overall average. Grand average of hydropathy (GRAVY) values ranged from −0.17 to −0.355, indicating that proteins are hydrophobic (**Table 3**). Because of this characteristic, proteins will fold correctly to maintain their stability and biological activity. The negative values of GRAVY indicates that proteins are non-polar and positive values of GRAVY indicate that proteins are polar. In *S. bicolor*, the peptides of 20S proteasome are rich in five amino acids (alanine, serine, glutamic acid, glycine, and leucine ranging from 8.93% to 12.22%). All these amino acids are involved in different biological functions in 20S proteasome (**Supplementary Table 6**). Five amino acids (arginine, phenylalanine, tyrosine, leucine, and glutamic acid) mainly provide catalytic sites for peptidase activity (**Supplementary Table 10**).

**Table 3:** Physicochemical properties of 20 SbPA/SbPB proteins associated with proteasomal degradation pathway.

S. no.	Name of protein	Molecular weight (D)	Theoretical pI	Negatively charged residues	Positively charged residues	Instability index	Aliphatic index	Hydropathy (GRAVY)	Stability
1	SbPAA1	27,415.26	6.1	34	31	35.97	83.66	−0.235	Stable
2	SbPAB1	25,867.33	5.2	28	23	36.73	86.68	−0.221	Stable
3	SbPAB2	25,864.35	5.5	28	24	35.05	88.77	−0.227	Stable
4	SbPAC1	26,995.74	6.43	25	24	49.39	85.16	−0.163	Unstable
5	SbPAD1	27,288.00	7.72	34	35	36.98	86.63	−0.355	Stable
6	SbPAE1	25,978.29	4.72	37	21	42.62	87.26	−0.221	Unstable
7	SbPAE2	25,977.31	4.76	36	21	43.43	87.26	−0.221	Unstable
8	SbPAF1	29,725.55	5.4	35	29	46.1	79.22	−0.278	Unstable
9	SbPAG1	22,375.5	8.26	23	25	34.96	85.05	−0.219	Stable
10	SbPAG2	27,237.08	5.92	34	30	31.38	90.88	−0.169	Stable
11	SbPBA1	26,207.57	5.71	25	19	29.41	85.18	−0.125	Stable
12	SbPBA2	26,277.71	5.71	25	19	29.41	85.18	−0.114	Stable
13	SbPBB1	29,220.24	6.7	28	27	24.54	78.21	−0.17	Stable
14	SbPBB2	29,185.22	6.24	30	27	25.17	80	−0.149	Stable
15	SbPBC1	22,854.22	5.12	27	21	30.83	87.45	−0.039	Stable
16	SbPBC2	22,855.25	5.16	26	21	34.26	86.52	−0.067	Stable
17	SbPBD1	23,184.71	5.59	28	24	23.34	100.76	0.053	Stable
18	SbPBE1	30,218.16	5.56	31	26	36.76	71.5	−0.162	Stable
19	SbPBF1	23,820.03	5.63	25	22	33.62	85.57	−0.128	Stable
20	SbPBG1	28,837.69	7.76	27	28	29.51	76.77	−0.327	Stable

#### 3.2.2 Functional domains and motifs of SbPA and SbPB proteins

The logo of 10 distinct motifs and the associated amino acids identified in the protein sequences of SbPA and SbPB proteins were given (**Supplementary Figure 4**). In **Supplementary Table 7**, the specifics of these proteins’ sequences, e-values, and functions are listed. These 20 motifs available in the database were not previously documented in rice and Arabidopsis (Parmentier et al., 1997; Fu et al., 1998; Sassa et al., 2000).

Recently, Sharma et al. (2022) reported 20 motifs in wheat. Individual motifs ranged in length from 15 aa (motif 8) to 50 aa (motifs 7 and 9). In cellular protein catabolic processes, the roles of motifs 2, 3, 4, 5, and 7 have been linked to proteolysis (https://www.ebi.ac.uk/interpro/result/InterProScan/iprscan5-R20211209-091508-0331-1308065-p2m/). The remaining five motifs need to be molecularly characterized because they were found to be novel. SbPA and SbPB proteins are anticipated to be in the nucleus and cytoplasm, as previously found in research for eukaryotes (Fu et al., 1998). One distinct α-type (1-7) or β-type (1-7) domain is present in each of the 20 SbPA and SbPB proteins. Ten of these SbPA/SbPB proteins have a single α/β-type domain (**Supplementary Table 5**).

In species like yeast, Arabidopsis, and rice, similar data on the 20S proteasome for individual and subunit proteins have not been provided (Groll et al., 1997; Parmentier et al., 1997; Fu et al., 1998; Sassa et al., 2000).

#### 3.2.3 Multiple sequence alignment and conserved amino acids

The OsPAE1 resembled 98.3% with SbPAE1 whereas OsPAF1 matched 89.55% with SbPAF1 (**Supplementary Table 8**). The OsPBC2 resembled 96.57% with SbPBC2 whereas OsPBE1 matched 83.33% with SbPBE1 (**Supplementary Table 9**). The similarity of all genes of *S. bicolor* with *O. sativa* was more than 80%. A high similarity of amino acids was observed between α- and β-subunits of *S. bicolor* and rice. A total of 5 aa of α-subunits and 8 aa of β-subunits were found to be highly conserved among *S. bicolor* and rice (**Supplementary Figures 5A, B**). These 5 (α-subunits) and 8 (β-subunits) residues also had the highest MI (**Supplementary Figures 6**, **7**).

#### 3.2.4 Subcellular localization and function

The proteasome complex includes the proteins SbPA and SbPB. These SbPA and SbPB proteins are found in the nucleus and cytoplasm These proteins may function in molecular processes such as response to zinc ions, proteasomal ubiquitin-independent protein metabolic processes, proteasome complex, proteasome core complex, proteasome core complex alpha-subunit complex, endopeptidase complex, and threonine-type endopeptidase activity, threonine-type peptidase activity, proteasome, peptidase complex, and response to metal ions, according to GO analysis and functional annotation (**Supplementary Figure 8**). We have performed protein–protein interaction network analysis to know the unknown functions of proteasomal proteins. We found 20 peptide nodes that correspond to number of gene sequences (**Supplementary Figure 9**). Based on the protein networks and their functional roles, we can say that the retrieved peptides show different types of functions such as ubiquitination, proteolysis, nitrogen metabolism, catabolic and anabolic processes, and endopeptidase and threonine peptidase activity (**Supplementary Table 10**).

#### 3.2.5 Secondary and tertiary structures

**Figure 5:**
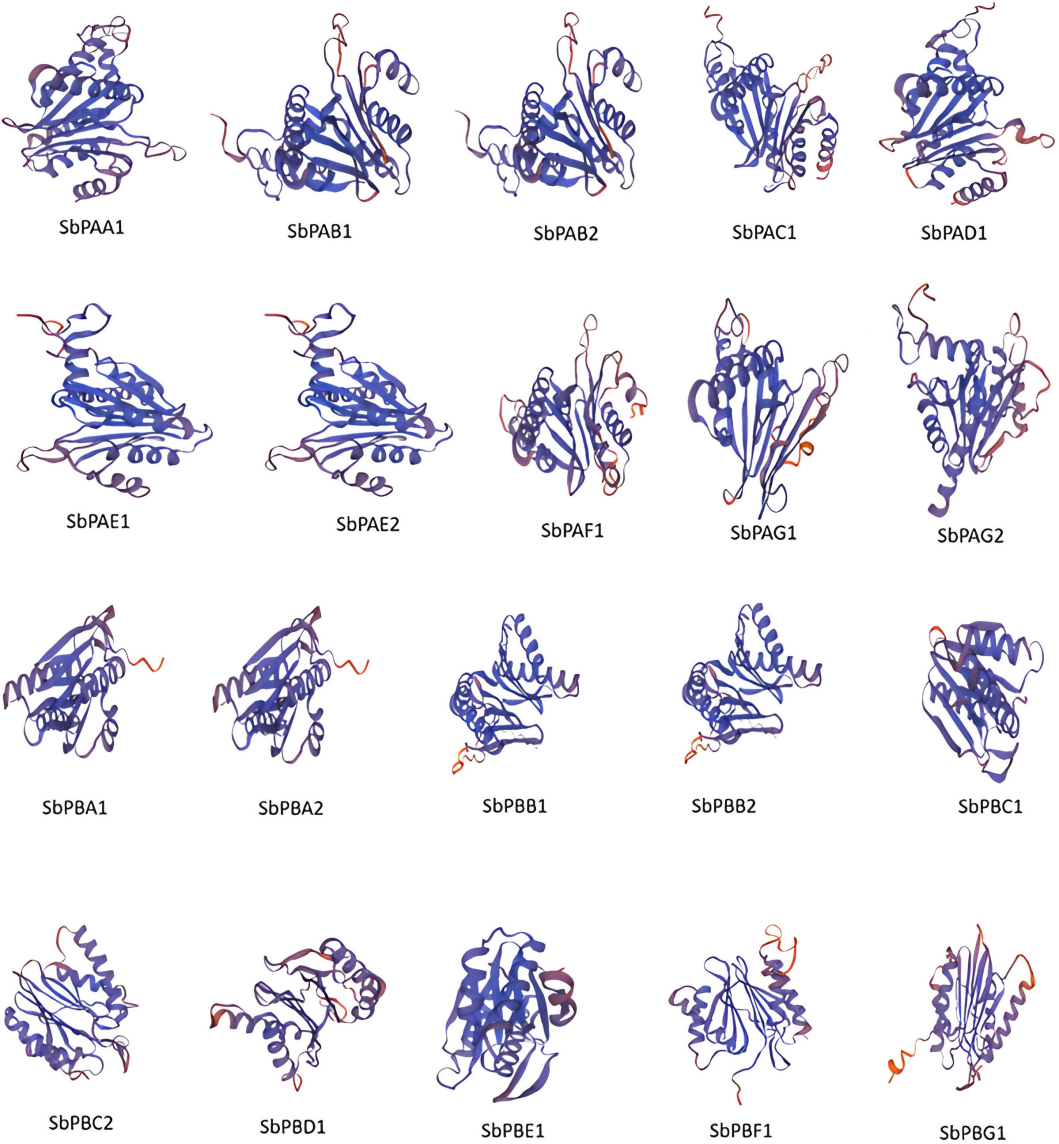
The 3D structures of 20 proteins encoded by *SbPA* and *SbPB* genes of 20S proteasome family in *Sorghum bicolor*. In all the 20 proteins, spirals represent helices, broad strips with arrowhead represent β-pleated sheets, and thin loops represent coil.

The secondary structures of all the proteins were compared. The secondary structures were found to be dominated by the α-helix followed by random coils, extended β-strand and turns for all sequences. The random coils created the irregular structural areas that allow polypeptide chains to fold in a distinctive manner (**Supplementary Table 11**). The SbPA and SbPB proteins tend to form highly stable structures. Only six (30%) SbPA and SbPB proteins with similarities ranging from 29.81% to 54.98% to the matching rice template were selected to determine *in silico* 3D structures. For these 20 proteins, the Global Model Quality Estimation (GMQE) ranged from 0.73 to 0.82. A high-grade protein model is suggested by this. The Q-mean value varied between 0.74 ± 0.05 and 0.80 ± 0.06. The protein model and the reference (rice) proteins were similar to varying degrees (60.57%). The 3D–1D score (found using verify 3D) ranged from 78.22% to 97.12%, and the quality factor (calculated using ERRAT) ranged from 86.1878 to 97.8355. (**Supplementary Table 12**). The modeling of the three-dimensional structure of the proteins was performed by the modeling program Swiss-Model and the modeled structures are shown in **Figure 5**. PROCHECK server analysis of the modeled protein revealed a varied percentage of residues under the most favored (85%–97%), generously allowed (0.5%–1.4%), additionally allowed (2.9%–14.6%), and disallowed regions (0.4%–1.0%), indicating that the predicted models were of excellent geometry and were accepted for further analysis (Laskowski et al., 1993; Kumar et al., 2018; Batra et al., 2019). Out of these 20 proteasomal proteins, 15 proteins have over 90% of their amino acid residues in energetically favored regions (**Supplementary Figure 10**). These proteins’ predicted 3D architectures give researchers a starting point for deciphering their molecular functions.

#### 3.2.6 Alignment and functional annotation of 3D structures

The 3D protein structures of *S. bicolor* were superimposed (using the least amount of energy) onto the matching 3D protein structures of rice reference proteins (**Supplementary Figure 11**). Six proteins’ 3D structures (SbPAB2, SbPAG2, SbPBA1, SbPBA2, SbPBB1, and SbPAD1) exhibited 1.3% to 3.07% resemblance to the matching OsPAA1 protein’s 3D structure, with an RMSD of 0 Å (**Supplementary Table 13**).

### 3.3 Phylogenetic analysis

**Figure 6:**
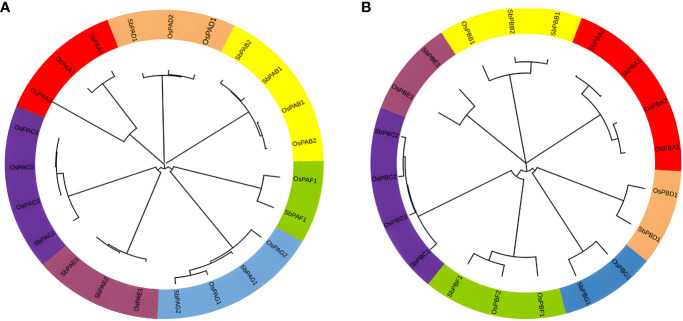
Phylogenetic tree constructed using protein sequences of **(A)** α-subunits and **(B)** β-subunits belonging to plant species, *O. sativa* and *S. bicolor*. Seven different colors in the tree represent seven different clades.

To create a phylogenetic tree, the amino acid sequences of *S*. *bicolor* and rice’s PA and PB subunits were used individually (**Figure 6**). Each of the phylogenetic trees consisting of seven specific clades was found (**Figures 6A, B**). Meme results showed that motifs 1, 2, 3, 4, 6, and 8 are most conserved in the alpha domain, whereas motifs 4, 3, and 2 are most conserved in the beta domain of proteasome. The conserved motifs and these clades were similar (**Figure 2**). The findings of seven clades in the present study may have numerous taxonomic applications (Fürstenberg-Hägg et al., 2013). It is interesting to note that seven clades in the phylogenetic tree were generated by the orthologs of three species that belong to distinct α- and β-subunits. A minimum of 7 and a maximum of 10 orthologs generated clades in the α-subunit tree. A minimum of 7 and a maximum of 13 orthologs generated clades in the β-subunit tree as well. Similar results were observed for rice, yeast, and Arabidopsis proteins (Fu et al., 1998; Sassa et al., 2000). These results revealed a higher degree of similarity between the α- and β-subunits of *S. bicolor* and the comparable subunits of rice. The results also suggested that 20S proteasome genes of *S. bicolor* are orthologous to rice genes.

### 3.4 Differential gene expression pattern of 20S proteasome genes

Gene expression patterns under normal and stressful circumstances were studied. Under normal conditions, we examined tissue-specific and development-specific expression. We also observed cold, drought- and hormone responses under abiotic stress.

#### 3.4.1 Tissue-specific expression

**Figure 7:**
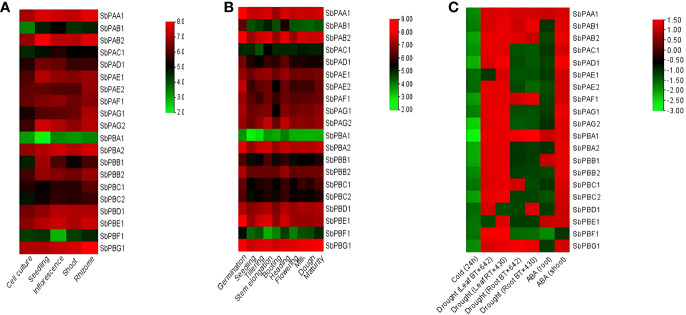
**(A)** Tissue-specific, **(B)** development-specific expression profile of *SbPA* and *SbPB* genes in *S. bicolor* under normal conditions, and **(C)** under abiotic stress. The expression data are represented in the form of fold values.

It was observed that different tissues (rhizome, shoot, inflorescence, seedling, and cell culture) showed variation in expression of 20S proteasomal genes. The rhizome showed the highest expression, which was then followed by the inflorescence, seedling, shoot, and cell culture (**Figure 7A**).

The expression analysis placed the 20 genes in three categories: (i) Higher expression: 16, 15, 14, 13, and 12 genes showed higher expression with six- to eightfold values in different organs, i.e., rhizome, inflorescence, seedling, shoot, and cell culture, respectively. (ii) Moderate expression: 4, 4, 3, 3, and 2 genes showed moderate expression with four- to sixfold values in different organs, i.e., seedling, shoot, inflorescence, cell culture, and rhizome, respectively. (iii) Lower expression: 5, 3, 2, 2, and 2 genes showed low expression with two- to fourfold values in different organs, i.e., cell culture, shoot, inflorescence, seedling, and rhizome, respectively (**Figure 7A**).

#### 3.4.2 Differential gene expression during plant development

It was observed that various developmental stages (germination, seedling, tillering, stem elongation, booting, heading, flowering, milk, dough, and maturity stages) showed variation in expression of 20S proteasomal genes (**Figure 7B**).

The development-specific expression analysis placed the 20 genes in two categories of higher and medium expression. A total of 19 genes showed higher expression with four- to eightfold values in different stages (germination, seedling, tillering, stem elongation, booting, heading, flowering, milk, dough, and maturity stages). Only one gene (*SbPBA1*) showed medium expression with zero- to fourfold values in different stages (germination, seedling, tillering, stem elongation, booting, heading, flowering, milk, dough, and maturity stages) (**Figure 7B**).

#### 3.4.3 Gene expression under abiotic stresses

##### 3.4.3.1 Cold stress

In cold stress, all genes were found to be downregulated. At 24 h, one gene (*SbPBA1*) showed higher expression, i.e., −2.80, one gene (*SbPAG2*) showed high to medium expression, i.e., −2.20 to −2.60, and five genes (*SbPAB2*, *SbPAD1*, *SbPBA2*, *SbPBB1*, and *SbPBC2*) showed medium expression, i.e., −2.0 to −2.20. In the same interval, all the remaining genes showed their expression in the range of −1.40 to −1.80, i.e., very poor expression. (**Figure 7C**).

##### 3.4.3.2 Drought stress

Under drought stress, two samples of each leaf (BT×642 and RT×430) and root (BT×642 and RT×430) were studied at pre-flowering stage for expression of 20S proteasome genes. The expression results showed downregulation (0.0 to −1.50-fold) of two genes (*SbPAE1* and *SbPBE1*) and one gene (*SbPBD1*) in BT x 642 and RT x 430 leaf samples, respectively. The remaining genes showed upregulation (0.0- to 1.50-fold) in their expression in both leaf samples. However, seven genes were found to be upregulated in each BT x 642 (*SbPAA1*, *SbPAB1*, *SbPAF1*, *SbPBC1*, *SbPAB2*, *SbPBA1*, and *SbPBG1*) and RT x 430 (*SbPAA1*, *SbPAB1, SbPAF1*, *SbPBD1*, *SbPAB2*, *SbPBA1*, and *SbPBG1*) root sample and their expression ranged from 0.0 to 1.50. All the remaining genes were found to be downregulated with an expression range of 0.0 to −2.0 in both root samples (**Figure 7C**).

##### 3.4.3.3 Hormone stress

Under abscisic acid (ABA) stress, 19 genes whose expression ranged from 0.0 to 1.50 were found upregulated, and one gene (*SbPBF1*) was found downregulated (0.0 to −2.0) in shoots. In roots, four genes (*SbPAA1*, *SbPBA1*, *SbPBB1*, and *SbPBE1*) whose expression ranged from 0.0 to 1.50 were upregulated and all the remaining genes showed downregulation (0.0 to −2.0) (**Figure 7C**).

**Figure 8:**
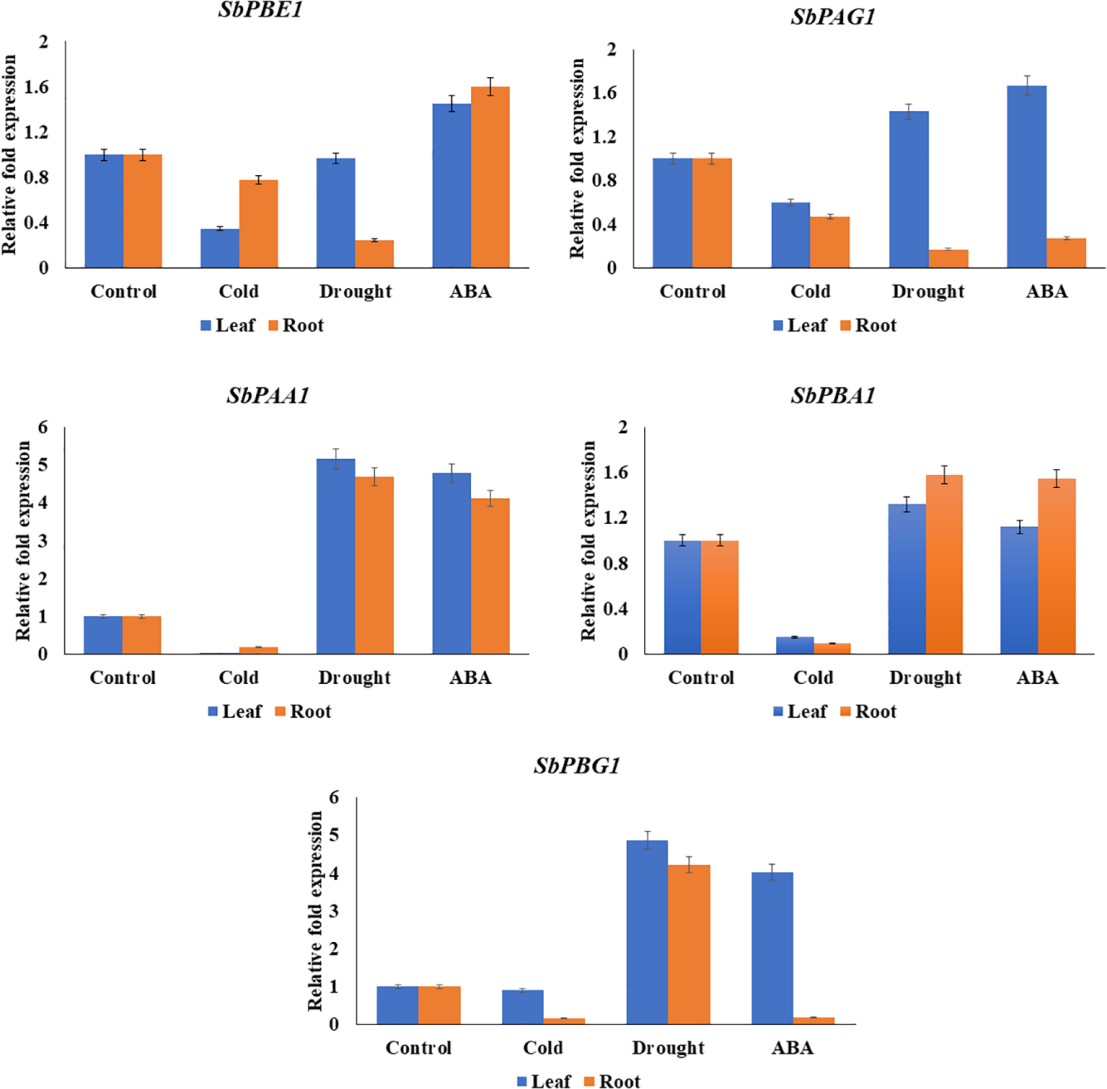
qRT-PCR expression levels of *SbPBA1*, *SbPBE1*, *SbPBG1, SbPAA1*, and *SbPAG1* genes under abiotic stresses (cold, drought, and ABA hormone) in *S. bicolor*.

The qRT-PCR analysis of candidate genes (*SbPBA1*, *SbPAA1*, *SbPBG1, SbPBE1*, and *SbPAG1*) was performed using gene-specific primers, listed in **Supplementary Table 14**. The genes showed similarity with *in silico* results. An increased expression of *SbPBA1* gene under drought and ABA treatments was found, i.e., 1.32- and 1.12-fold in leaves and 1.58- and 1.55-fold in roots, as compared to the control conditions. *SbPBE1* gene was found upregulated only under ABA stress in both leaf (1.45-fold) and root (1.6-fold) tissues. Downregulation of this gene was found under cold and drought stress. An upregulation in expression of *SbPAG1* under drought (1.43-fold) and ABA (1.67-fold) stress in leaf tissues but not in roots was also observed. An increased expression of *SbPAA1* gene under drought and ABA treatments was also found, i.e., 5.17- and 4.78-fold in leaves and 4.69- and 4.12-fold in roots, as compared to the control conditions. In leaves, under drought and ABA stress, *SbPBG1* showed increased expression of 4.02- to 4.87-fold. These five genes studied for qRT-PCR expression showed their downregulation under cold treatment (**Figure 8**).

## 4 Discussion

The present investigation revealed 20 proteasomal genes in *S. bicolor*. These genes were organized into seven different α and seven different β types of 20S proteasome family. In the majority of the SbPA and SbPB genes the structural pattern of exon and intron was found to be similar, although in some cases, this similarity pattern deviates. This deviation may be due to loss or gain of intron during gene evolution (Rogozin et al., 2003; Yu et al., 2019). In cDNA sequences, major differences can be due to size and number of introns present in SbPA and SbPB genes. Moreover, differences in the length of cDNA sequences may be due to variation in the length of UTRs present on the borders of cDNA. The phylogenomic analysis of 20 genes revealed that there are seven clades of each α (SbPAA-SbPAG) and β (SbPBA-SbPBG). An uneven distribution of *SbPA* and *SbPB* genes on 10 chromosomes and this may be due to gene duplication or gene loss (Li et al., 2015; Clavijo et al., 2017; Yu et al., 2019). Gene duplication is a random and frequent process/event that occurs by either tandem or block duplication mechanisms. The duplication events are helpful in understanding the expansion mechanism of 20S proteasomal family genes. Eleven (55%) of the 20 genes included a total of 22 SSRs. This suggests that only a small fraction of the genes in a gene family include SSRs. The structural and functional characteristics of SSR have been identified in a large number of genes (Gupta and Rustgi, 2004; Li et al., 2004; Varshney et al., 2005). In Sorghum, the simple sequence repeats (SSRs) were mapped by Taramino et al. (1997). The SSRs found in the genes encoding the 20S proteasome’s α- and β-subunits can be exploited to provide useful/functional markers for marker-assisted selection. These biomarkers can be employed to increase plant system tolerance to biotic and abiotic challenges. The lack of transposable and retro elements in the genes under investigation implied that the 20S proteasome family is not expressed in *S*. *bicolor*. Promoter analysis revealed that there are a wide range of cis regulatory elements that mediate transcriptional regulation of 20S proteasomal genes. The investigation employed the 1,500-bp 5’ upstream of the promoter sequence. The development and stress response in Sorghum were found to be regulated by the consensus cis regulatory elements that were found to span the promoter of 20 proteasomal genes. A typical cis-acting element in promoter and enhancer regions is the CAAT-box. All families of transcription factors included it. Low-temperature responsiveness (LTR), drought responsiveness (MBS), and defense and stress responsiveness (TC rich repeats) are the recognized stress-related motifs. Similar to this, light responsiveness (LAMP element, MRE, TCT-motif, TCCC-motif, I-box, Sp1, GT1-motif, GTGGC-motif, GATA-motif, Box 4, Box II, ATCT-motif, PcCMA2c, chsCMA2a) and zein metabolism (O2 site) are development-related characteristics. Auxin responsiveness (AuxRR-core), abscisic acid responsiveness (ABRE), ethylene responsiveness, GA3 response (G-box, P-box, GARE-motif, and TATC-box), salicylic acid responsiveness (TCA element), and MeJA responsiveness are a few more hormone-responsive motifs that have been discovered (CGTCA and TGACG motifs). Small non-coding RNAs called miRNAs play regulatory roles in cells at the post-transcriptional and translational levels. These cause result gene targets to deteriorate (Budak and Zhang, 2017). Upon induction, the microRNA sbi-miR396d induced drought tolerance in Arabidopsis by targeting growth-regulating factors coordinating cell division and differentiation that impact leaf development and orientation (Jones-Rhoades and Bartel, 2004).

Digital expression analysis of the 20S proteasome genes in plant systems was previously studied (Fu et al., 1998; Li et al., 2015). Many biological processes, including plant development and reactions to various biotic and abiotic stressors, include 20S proteasome genes (Xu and Xue, 2019). These actions are a result of impulses from various signaling molecules that control plant growth under various stress conditions (Livneh et al., 2016). Numerous indications point to the possibility that hormone signals have an impact on the 20S proteasome gene’s expression (Kurepa et al., 2009). A number of genes contributing biotic and abiotic stress tolerance have been identified by studying UPS genetics in different crops, for example, TaFBA1’s role in heat tolerance (Li et al., 2018), the role of heat shock proteins in the breakdown of toxic and misfolded proteins (Awasthi and Wagner, 2005), and its participation in a number of human disorders (Bozaykut et al., 2020). This is the first investigation on the expression of 20S proteasome genes in *S. bicolor* under both normal and stressful circumstances. A publicly accessible transcriptome database was used for this.

Hoffmann and Rooney (2019) noted that the production of sorghum is impacted by both abiotic [drought, temperature (heat or cold), and soil fertility and/or composition (specifically soil pH, micronutrients, or fertility)] and biotic {insects like the sugarcane aphid (*Melanaphis sacchari*) and plant pathogens like stalk rot (*Macrophomina phaseolina*), head worm [*Helicoverpa zea* (Boddie)], midge [*Contarinla sorghicola* (Coquillett)], and green bug [*Schizaphis graminum* (Rondani)]}. Roozeboom and Prasad (2019) reported that Sorghum is a remarkably resilient plant, with the ability to compensate environmental stresses, insecticidal diseases, and nutrient availability. In comparison to wild types (with bloom phenotype), Jenks et al. (1994) discovered that leaves of a bloomless Sorghum mutant with thinner cuticles were more vulnerable to the fungi *Exserohilum turcicum* (Pass.) and *Puccinia purpurea* (eke) in the field. According to Burow et al. (2007), the bloom gene may significantly contribute to Sorghum’s overall drought resistance. The upregulation of *SbPBA1*, *SbPAA1*, *SbPBG1*, *SbPBE1*, and *SbPAG1* under ABA and drought stress provide an insight into its involvement in abiotic stress. No expression was observed for cold stress of these genes indicating their non-involvement (**Figure 8**). Transcript levels of *Glycine max UBC2* (*GmUBC2*) and *Arabidopsis UBC32* (*AtUBC32*) are upregulated in response to drought and/or salt stress (Zhou et al., 2010; Wan et al., 2011; Cui et al., 2012). Transgenic Arabidopsis plants overexpressing *Vigna radiata* UBC1 (VrUBC1), AhUBC2, or GmUBC2 are more tolerant to drought stress (Zhou et al., 2010; Wan et al., 2011; Chung et al., 2013). In plants, UPS components contribute to ABA-dependent responses to abiotic stresses by regulating ABA biosynthesis (through XERICO and PUB44) and ABA signaling (through ABA receptors PYL/PYR/RCAR, suppressors PP2C and transcription factors ABFs/ABIs) (Xu and Xue, 2019). According to a recent study, the deletion of PBE1, a β5 subunit, had a significant impact on how proteasomes assembled when subjected to salt stress (Han et al., 2019), proving that PBE1 is necessary for complete proteasome assembly. Additionally, PBE1 was discovered to reduce the transcription factor ABI5’s protein accumulating activity, altering ABA-mediated salt stress signaling in plants (Han et al., 2019). The function of these above-mentioned genes that show differential expression may be planned for future studies in different breeding programs in order to develop sorghum cultivar resistant to various stresses.

The ubiquitin/20S proteasome system (UPS), which works after external stimuli, allows plants to modify their proteomes in response to their environment in order to develop and survive. Only a few genes and their functions have been fully understood to date out of the many genes that encode UPS components. It will be easier to understand the molecular regulatory mechanisms that underlie plant responses to environmental stimuli at the protein level with further identification of distinct substrates of proteasomal subunits and proteasome regulators, which will also help to provide practical strategies to increase crop tolerance to both biotic and abiotic stresses. Homologous or heterologous gene expression-based genetic engineering can produce genotypes that perform better under environmental stress (Han et al., 2019). It may be attempted to boost stress tolerance in crops including rice, maize, sorghum, and wheat by overexpressing particular UPS components that act as positive regulators of various types of stress tolerance.

## 5 Conclusion

In the current study, the 10 SbPA and 10 SbPB genes of the 20S proteasome were discovered which serves as genetic tools for functional analysis of 20S proteasome in *S. bicolor*. Orthology of *SbPA* and *SbPB* genes with rice was inferred and identified. The proteins that the *SbPA* and *SbPB* genes encode have a full-length 3D model and are capable of imparting distinct proteolytic and biological functions to the 20S complex. Under typical abiotic stress, it was discovered that various *S. bicolor* organs expressed a number of *SbPA* and *SbPB* genes. In response to abiotic stressors and in the development of many plant organs, the 20S proteasome gene is crucial. In this approach, the current study offers a wealth of knowledge that can be applied to the creation of *S. bicolor* cultivars that are climatically adaptable.

## Data availability statement

The original contributions presented in the study are included in the article/**Supplementary Material**, further inquiries can be directed to the corresponding author.

## Author contributions

VM: Investigation, Software, Supervision, Writing – original draft, Writing – review & editing. FI: Formal Analysis, Software, Writing – original draft. YS: Conceptualization, Data curation, Formal Analysis, Software, Validation, Writing – original draft, Writing – review & editing. VK: Data curation, Formal Analysis, Methodology, Software, Writing – original draft, Writing – review & editing. PM: Data curation, Formal Analysis, Methodology, Software, Writing – review & editing. PB: Conceptualization, Data curation, Formal Analysis, Methodology, Software, Writing – original draft, Writing – review & editing. KS: Conceptualization, Investigation, Project administration, Software, Supervision, Writing – review & editing. RM: Conceptualization, Formal Analysis, Investigation, Project administration, Supervision, Writing – review & editing. AH: Data curation, Formal Analysis, Funding acquisition, Methodology, Software, Writing – review & editing. GA-Q: Data curation, Formal Analysis, Funding acquisition, Methodology, Project administration, Software, Supervision, Writing – review & editing. EA: Data curation, Funding acquisition, Methodology, Software, Writing – review & editing. UK: Conceptualization, Data curation, Formal Analysis, Investigation, Methodology, Project administration, Software, Supervision, Validation, Writing – original draft, Writing – review & editing.

## References

[B1] Awasthi N. Wagner B. J. (2005). Upregulation of heat shock protein expression by proteasome inhibition: an antiapoptotic mechanism in the lens. Invest. Ophthalmol. Visual Sci. 46 (6), 2082–2091. doi: 10.1167/iovs.05-0002 15914627

[B2] Bailey T. L. Boden M. Buske F. A. Frith M. Grant C. E. Clementi L.. (2009). MEME SUITE: tools for motif discovery and searching. Nucleic Acids Res. 37, 202–208. doi: 10.1093/nar/gkp335 PMC2703892 19458158

[B3] Batra R. Agarwal P. Tyagi S. Saini D. K. Kumar V. Kumar A.. (2019). A study of CCD8 genes/proteins in seven monocots and eight dicots. PloS One 14 (3), e0213531. doi: 10.1371/journal.pone.0213531 30861026 PMC6413960

[B4] Bozaykut P. Sozen E. Kaga E. Ece A. Ozaltin E. Bergquist J.. (2020). HSP70 inhibition leads to the activation of proteasomal system under mild hyperthermia conditions in young and senescent fibroblasts. Oxid. Med. Cell. longevity 2020, 1–10. doi: 10.1155/2020/9369524 PMC7064868 32190179

[B5] Budak H. Zhang B. (2017). MicroRNAs in model and complex organisms. Funct. Integr. Genomics 17 (2), 121–124. doi: 10.1007/s10142-017-0544-1 28220336

[B6] Burow G. Franks C. Payton P. Xin Z. (2007). Genetic Mapping and Characterization of an Epicuticular Wax (Bloom) Gene, Sb Bl, in Sorghum bicolor L. (Moench) (Soils and Environment, New Orleans, Louisiana: International Annual Meetings on A Century of Integrating Crops).

[B7] Cai Y. M. Yu J. Ge Y. Mironov A. Gallois P. (2018). Two proteases with caspase-3-like activity, cathepsin B and proteasome, antagonistically control ER-stress-induced programmed cell death in Arabidopsis. New Phytol. 218 (3), 1143–1155. doi: 10.1111/nph.14676 28675441 PMC5947621

[B9] Chen N. (2004). Using Repeat Masker to identify repetitive elements in genomic sequences. Curr. Protoc. Bioinf. 5 (1), 4–10. doi: 10.1002/0471250953.bi0410s05 18428725

[B8] Chen S. Jia H. Wang X. Shi C. Wang X. Ma P.. (2020). Hydrogen sulfide positively regulates abscisic acid signaling through persulfidation of SnRK2. 6 in guard cells. Mol. Plant 13, 732–744. doi: 10.1016/j.molp.2020.01.004 31958520

[B10] Chow C.-N. Zheng H.-Q. Wu N.-Y. Chien C.-H. Huang H.-D. Lee T.-Y.. (2016). PlantPAN 2.0: an update of plant promoter analysis navigator for reconstructing transcriptional regulatory networks in plants. Nucleic Acids Res. 44, 1154–1160. doi: 10.1093/nar/gkv1035 PMC4702776 26476450

[B11] Chung E. Cho C. W. So H. A. Kang J. S. Chung Y. S. Lee J. H. (2013). Overexpression of VrUBC1, a mung bean E2 ubiquitin-conjugating enzyme, enhances osmotic stress tolerance in Arabidopsis. PloS One 8, e66056. doi: 10.1371/journal.pone.0066056 23824688 PMC3688854

[B12] Clavijo B. J. Venturini L. Schudoma C. Accinelli G. G. Kaithakottil G. Wright J.. (2017). An improved assembly and annotation of the allohexaploid wheat genome identifies complete families of agronomic genes and provides genomic evidence for chromosomal translocations. Genome Res. 27 (5), 885–896. doi: 10.1101/gr.217117.116 28420692 PMC5411782

[B13] Cui F. Liu L. Zhao Q. Zhang Z. Li Q. Lin B.. (2012). Arabidopsis ubiquitin conjugase UBC32 is an ERAD component that functions in brassinosteroid-mediated salt stress tolerance. Plant Cell 24, 233–244. doi: 10.1105/tpc.111.093062 22214659 PMC3289556

[B14] Dai X. Zhuang Z. Zhao P. X. (2018). psRNATarget: a plant small RNA target analysis server. Nucleic Acids Res. 46, 49–54. doi: 10.1093/nar/gky316 PMC6030838 29718424

[B16] Dhankher O. P. Foyer C. H. (2018). Climate resilient crops for improving global food security and safety. Plant Cell Environ. 41 (5), 877–884. doi: 10.1111/pce.13207 29663504

[B17] Dielen A. S. Sassaki F. T. Walter J. Michon T. Menard G. Pagny G.. (2011). The 20S proteasome α5 subunit of Arabidopsis thaliana carries an RNase activity and interacts in planta with the lettuce mosaic potyvirus HcPro protein. Mol. Plant Pathol. 12 (2), 137–150. doi: 10.1111/j.1364-3703.2010.00654.x 21199564 PMC6640220

[B18] Eisenberg D. Lüthy R. Bowie J. U. (1997). [20] VERIFY3D: assessment of protein models with three-dimensional profiles. Methods enzymology 277, 396–404). doi: 10.1016/S0076-6879(97)77022-8 9379925

[B19] Finley D. (2009). Recognition and processing of ubiquitin-protein conjugates by the proteasome. Annu. Rev. Biochem. 78, 477–513. doi: 10.1146/annurev.biochem.78.081507.101607 19489727 PMC3431160

[B20] Fu H. Doelling J. H. Arendt C. S. Hochstrasser M. Vierstra R. D. (1998). Molecular organization of the 20S proteasome gene family from *Arabidopsis thaliana* . Genetics 149 (2), 677–692. doi: 10.1093/genetics/149.2.677 9611183 PMC1460176

[B21] Fürstenberg-Hägg J. Zagrobelny M. Bak S. (2013). Plant defense against insect herbivores. Int. J. Mol. Sci. 14 (5), 10242–10297. doi: 10.3390/ijms140510242 23681010 PMC3676838

[B22] Ge S. X. Jung D. Yao R. (2020). ShinyGO: a graphical gene-set enrichment tool for animals and plants. Bioinformatics 36, 2628–2629. doi: 10.1093/bioinformatics/btz931 31882993 PMC7178415

[B23] Geourjon C. Deleage G. (1995). SOPMA: significant improvements in protein secondary structure prediction by consensus prediction from multiple alignments. Bioinformatics 11 (6), 681–684. doi: 10.1093/bioinformatics/11.6.681 8808585

[B24] Glickman M. H. Ciechanover A. (2002). The ubiquitin-proteasome proteolytic pathway: destruction for the sake of construction. Physiol. Rev. 82, 373–428. doi: 10.1152/physrev.00027.2001 11917093

[B25] Groll M. Ditzel L. Löwe J. Stock D. Bochtler M. Bartunik H. D.. (1997). Structure of 20S proteasome from yeast at 2.4 Å resolution. Nature 386 (6624), 463–471. doi: 10.1038/386463a0 9087403

[B26] Gupta P. K. Rustgi S. (2004). Molecular markers from the transcribed/expressed region of the genome in higher plants. Funct. Integr. Genomics 4 (3), 139–162. doi: 10.1007/s10142-004-0107-0 15095058

[B27] Han J. J. Yang X. Wang Q. Tang L. Yu F. Huang X.. (2019). The β5 subunit is essential for intact 26S proteasome assembly to specifically promote plant autotrophic growth under salt stress. New Phytol. 221 (3), 1359–1368. doi: 10.1111/nph.15471 30346042

[B28] Hoffmann L. Jr. Rooney W. L. (2019). Sorghum improvement for yield. Sorghum: A State Art Future Perspetives 58, 31–46. doi: 10.2134/agronmonogr58.c2

[B29] Hruz T. Laule O. Szabo G. Wessendorp F. Bleuler S. Oertle L.. (2008). Genevestigator v3: a reference expression database for the metaanalysis of transcriptomes. Adv. Bioinf. 3, 1–5. doi: 10.1155/2008/420747 PMC2777001 19956698

[B30] Hu B. Jin J. Guo A. Y. Zhang H. Luo J. Gao G. (2015). GSDS 2.0: an upgraded gene feature visualization server. Bioinformatics 31, 1296–1297. doi: 10.1093/bioinformatics/btu817 25504850 PMC4393523

[B31] Jenks M. A. Joly R. J. Peters P. J. Rich P. J. Axtell J. D. Ashworth E. N. (1994). Chemically induced cuticle mutation affecting epidermal conductance to water vapour and disease susceptibility in Sorghum bicolor (L.) Moench. Plant Physiol. 105, 1239–1245. doi: 10.1104/pp.105.4.1239 12232280 PMC159454

[B32] Jones-Rhoades M. W. Bartel D. P. (2004). Computational identification of plant microRNAs and their targets, including a stress-induced miRNA. Mol. Cell 14 (6), 787–799. doi: 10.1016/j.molcel.2004.05.027 15200956

[B33] Kumar A. Sharma P. Gomar-Alba M. Shcheprova Z. Daulny A. Sanmartín T.. (2018). Daughter-cell-specific modulation of nuclear pore complexes controls cell cycle entry during asymmetric division. Nat. Cell Biol. 20 (4), 432–442. doi: 10.1038/s41556-018-0056-9 29531309 PMC6029668

[B34] Kumar V. Joshi S. Pant N. C. Sangwan P. Yadav A. N. Saxena A.. (2019). “Molecular approaches for combating multiple abiotic stresses in crops of arid and semi-arid region,” in Molecular approaches in plant biology and environmental challenges (Singapore: Springer), 149–170. doi: 10.1007/978-981-15-0690-1_8

[B35] Kumar V. Sharma H. Saini L. Tyagi A. Jain P. Singh Y.. (2022). Phylogenomic analysis of 20S proteasome gene family reveals stress-responsive patterns in rapeseed (Brassica napus L.). Front. Plant Sci. 4269. doi: 10.3389/fpls.2022.1037206 PMC9659873 36388569

[B36] Kurata N. Yamazaki Y. (2006). Oryzabase. An integrated biological and genome information database for rice. Plant Physiol. 140, 12–17. doi: 10.1104/pp.105.063008 16403737 PMC1326027

[B37] Kurepa J. Wang S. Li Y. Smalle J. (2009). Proteasome regulation, plant growth and stress tolerance. Plant Signaling Behav. 4 (10), 924–927. doi: 10.4161/psb.4.10.9469 PMC2801354 19826220

[B38] Laskowski R. A. MacArthur M. W. Moss D. S. Thornton J. M. (1993). PROCHECK: a program to check the stereochemical quality of protein structures. J. Appl. Crystallogr. 26 (2), 283–291. doi: 10.1107/S0021889892009944

[B39] Lescot M. Dehais P. Thijs G. Marchal K. Moreau Y. Van de Peer Y.. (2002). PlantCARE, a database of plant cis-acting regulatory elements and a portal to tools for in silico analysis of promoter sequences. Nucleic Acids Res. 30, 325–327. doi: 10.1093/nar/30.1.325 11752327 PMC99092

[B40] Letunic I. Bork P. (2016). Interactive tree of life (iTOL) v3: an online tool for the display and annotation of phylogenetic and other trees. Nucleic Acids Res. 44, 242–245. doi: 10.1093/nar/gkw290 PMC4987883 27095192

[B41] Letunic I. Doerks T. Bork P. (2009). SMART 6: recent updates and new developments. Nucleic Acids Res. 37, 229–232. doi: 10.1093/nar/gkn808 PMC2686533 18978020

[B44] Li Q. Wang W. Wang W. Zhang G. Liu Y. Wang Y.. (2018). Wheat F-box protein gene TaFBA1 is involved in plant tolerance to heat stress. Front. Plant Sci. 9, 521. doi: 10.3389/fpls.2018.00521 29740462 PMC5928245

[B42] Li X. M. Chao D. Y. Wu Y. Huang X. Chen K. Cui L. G.. (2015). Natural alleles of a proteasome α2 subunit gene contribute to thermotolerance and adaptation of African rice. Nat. Genet. 47 (7), 827–833. doi: 10.1038/ng.3305 25985140

[B43] Li Y. C. Korol A. B. Fahima T. Nevo E. (2004). Microsatellites within genes: structure, function, and evolution. Mol. Biol. Evol. 21 (6), 991–1007. doi: 10.1093/molbev/msh073 14963101

[B45] Livneh I. Cohen-Kaplan V. Cohen-Rosenzweig C. Avni N. Ciechanover A. (2016). The life cycle of the 26S proteasome: from birth, through regulation and function and onto its death. Cell Res. 26 (8), 869–885. doi: 10.1038/cr.2016.86 27444871 PMC4973335

[B47] Lu K. Wei L. Li X. Wang Y. Wu J. Liu M.. (2019). Whole-genome resequencing reveals Brassica napus origin and genetic loci involved in its improvement. Nat. Commun. 10 (1), 1–12. doi: 10.1038/s41467-019-09134-9 30858362 PMC6411957

[B46] Lu S. Wang J. Chitsaz F. Derbyshire M. K. Geer R. C. Gonzales N. R.. (2020). CDD/SPARCLE: the conserved domain database in 2020. Nucleic Acids Res. 48, 265–268. doi: 10.1093/nar/gkz991 PMC6943070 31777944

[B48] Lyons E. Freeling M. (2008). How to usefully compare homologous plant genes and chromosomes as DNA sequences. Plant J. 53, 661–673. doi: 10.1111/j.1365-313X.2007.03326.x 18269575

[B49] Muffato M. Louis A. Poisnel C. E. Crollius H. R. (2010). Genomicus. Bioinformatics 26 (8), 1119–1121. doi: 10.1093/bioinformatics/btq079 20185404 PMC2853686

[B50] Parmentier Y. Bouchez D. Fleck J. Genschik P. (1997). The 20S proteasome gene family in Arabidopsis thaliana. FEBS Lett. 416 (3), 281–285. doi: 10.1016/S0014-5793(97)01228-3 9373170

[B51] Proost S. Van Bel M. Sterck L. Billiau K. Van Parys T. Van de Peer Y.. (2009). PLAZA: a comparative genomics resource to study gene and genome evolution in plants. Plant Cell 21, 3718–3731. doi: 10.1105/tpc.109.071506 20040540 PMC2814516

[B52] Rensink W. A. Buell C. R. (2004). Arabidopsis to rice. Applying knowledge from a weed to enhance our understanding of a crop species. Plant Physiol. 135 (2), 622–629. doi: 10.1104/pp.104.040170 15208410 PMC514098

[B53] Rogozin I. B. Wolf Y. I. Sorokin A. V. Mirkin B. G. Koonin E. V. (2003). Remarkable interkingdom conservation of intron positions and massive lineage-specific intron loss and gain in eukaryotic evolution. Curr. Biol. 13 (17), 1512–1517. doi: 10.1016/S0960-9822(03)00558-X 12956953

[B54] Roozeboom K. L. Prasad P. V. (2019). Sorghum growth and development. Sorghum: A State Art Future Perspect. 58, 155–172. doi: 10.2134/agronmonogr58.c8

[B55] Saeki Y. (2017). Ubiquitin recognition by the proteasome. J. Biochem. 161, 113–124. doi: 10.1093/jb/mvw091 28069863

[B56] Sakai H. Lee S. S. Tanaka T. Numa H. Kim J. Kawahara Y.. (2013). Rice Annotation Project Database (RAP-DB): an integrative and interactive database for rice genomics. Plant Cell Physiol. 54, 6. doi: 10.1093/pcp/pcs183 PMC3583025 23299411

[B57] Sassa H. Oguchi S. Inoue T. Hirano H. (2000). Primary structural features of the 20S proteasome subunits of rice (Oryza sativa). Gene 250 (1-2), 61–66. doi: 10.1016/S0378-1119(00)00190-6 10854779

[B59] Sharma H. Batra R. Kumar S. Kumar M. Kumar S. Balyan H. S.. (2022). Identification and characterization of 20S proteasome genes and their relevance to heat/drought tolerance in bread wheat. Gene Rep. 27, 101552. doi: 10.1016/j.genrep.2022.101552

[B60] Smedley D. Haider S. Ballester B. Holland R. London D. Thorisson G.. (2009). BioMart–biological queries made easy. BMC Genomics 10 (1), 1–12. doi: 10.1186/1471-2164-10-22 19144180 PMC2649164

[B61] Song X. J. Huang W. Shi M. Zhu M. Z. Lin H. X. (2007). A QTL for rice grain width and weight encodes a previously unknown RING-type E3 ubiquitin ligase. Nat. Genet. 39, 623–630. doi: 10.1038/ng2014 17417637

[B62] Sun H. H. Fukao Y. Ishida S. Yamamoto H. Maekawa S. Fujiwara M.. (2013). Proteomics analysis reveals a highly heterogeneous proteasome composition and the post-translational regulation of peptidase activity under pathogen signaling in plants. J. Proteome Res. 12 (11), 5084–5095. doi: 10.1021/pr400630w 23991809

[B64] Sun Q. Miao C. Hanel M. Borthwick A. G. Duan Q. Ji D.. (2019). Global heat stress on health, wildfires, and agricultural crops under different levels of climate warming. Environ. Int. 128, 125–136. doi: 10.1016/j.envint.2019.04.025 31048130

[B63] Sun L. Yang Z. T. Song Z. T. Wang M. J. Sun L. Lu S. J.. (2013). The plant-specific transcription factor gene NAC 103 is induced by b ZIP 60 through a new cis-regulatory element to modulate the unfolded protein response in A rabidopsis. Plant J. 76 (2), 274–286. doi: 10.1111/tpj.12287 23869562

[B65] Sung M. K. Reitsma J. M. Sweredoski M. J. Hess S. Deshaies R. J. (2016). Ribosomal proteins produced in excess are degraded by the ubiquitin–proteasome system. Mol. Biol. Cell 27 (17), 2642–2652. doi: 10.1091/mbc.E16-05-0290 27385339 PMC5007085

[B66] Swarbreck D. Wilks C. Lamesch P. Berardini T. Z. Garcia-Hernandez M. Foerster H.. (2007). The Arabidopsis Information Resource (TAIR): gene structure and function annotation. Nucleic Acids Res. 36, 1009–1014. doi: 10.1093/nar/gkm965 17986450 PMC2238962

[B58] Szklarczyk D. Kirsch R. Koutrouli M. Nastou K. Mehryary F. Hachilif R.. (2023). The STRING database in 2023: protein–protein association networks and functional enrichment analyses for any sequenced genome of interest. Nucleic Acids Res. 51 (D1), D638–D646. doi: 10.1093/nar/gkac1000 36370105 PMC9825434

[B67] Taramino G. Tarchini R. Ferrario S. Lee M. (1997). Characterization and mapping of simple sequence repeats (SSRs) in Sorghum bicolor. Theor. Appl. Genet. 95 (1-2), 66–72. doi: 10.1007/s001220050533

[B68] Toufighi K. Brady S. M. Austin R. Ly E. Provart N. J. (2005). The Botany Array Resource: e-Northerns, expression angling, and promoter analyses. Plant J. 43, 153–163. doi: 10.1111/j.1365-313X.2005.02437.x 15960624

[B69] Varshney R. K. Graner A. Sorrells M. E. (2005). Genic microsatellite markers in plants: features and applications. Trends Biotechnol. 23 (1), 48–55. doi: 10.1016/j.tibtech.2004.11.005 15629858

[B70] Wan X. Mo A. Liu S. Yang L. Li L. (2011). Constitutive expression ofa peanut ubiquitin-conjugating enzyme gene in Arabidopsis confers improved water-stress tolerance through regulation of stress-responsive gene expression. J. Bioscience Bioengineering 111, 478–484. doi: 10.1016/j.jbiosc.2010.11.021 21193345

[B71] Wang G. Fan W. Ou M. Wang X. Qin H. Feng F.. (2019). Dek40 encodes a PBAC4 protein required for 20S proteasome biogenesis and seed development. Plant Physiol. 180 (4), 2120–2132. doi: 10.1104/pp.18.01419 31189659 PMC6670095

[B72] Xia L. Zou D. Sang J. Xu X. Yin H. Li M.. (2017). Rice Expression Database (RED): an integrated RNA-Seq-derived gene expression database for rice. J. Genet. Genomics 44, 235–241. doi: 10.1016/j.jgg.2017.05.003 28529082

[B73] Xu F. Q. Xue H. W. (2019). The ubiquitin-proteasome system in plant responses to environments. Plant Cell Environ. 42 (10), 2931–2944. doi: 10.1111/pce.13633 31364170

[B74] Yu X. Han J. Wang E. Xiao J. Hu R. Yang G.. (2019). Genome-wide identification and homoeologous expression analysis of PP2C genes in wheat (Triticum aestivum L.). Front. Genet. 10. doi: 10.3389/fgene.2019.00561 PMC6582248 31249596

[B75] Yu H. Matouschek A. (2017). Recognition of client proteins by the proteasome. Annu. Rev. Biophys. 46, 149–173. doi: 10.1146/annurev-biophys-070816-033719 28301771

[B76] Zhou G. A. Chang R. Z. Qiu L. J. (2010). Overexpression of soybean ubiquitin-conjugating enzyme gene GmUBC2 confers enhanced drought and salt tolerance through modulating abiotic stress-responsive gene expression inArabidopsis. Plant Mol. Biol. 72, 357–367. doi: 10.1007/s11103-009-9575-x 19941154 PMC2816239

