# Supplementary material for: Structural and functional characteristics and expression profile of the 20S proteasome gene family in Sorghum under abiotic stress
Source: Front Plant Sci. 2023 Nov 29;14:1287950. doi: 10.3389/fpls.2023.1287950 (PMC10716288; doi:10.3389/fpls.2023.1287950)
Supplement: Supplementary file 1 [file DataSheet_1.docx]

**Structural and functional characteristics and expression profile of 20S proteasome gene family in Sorghum under abiotic stress**

**
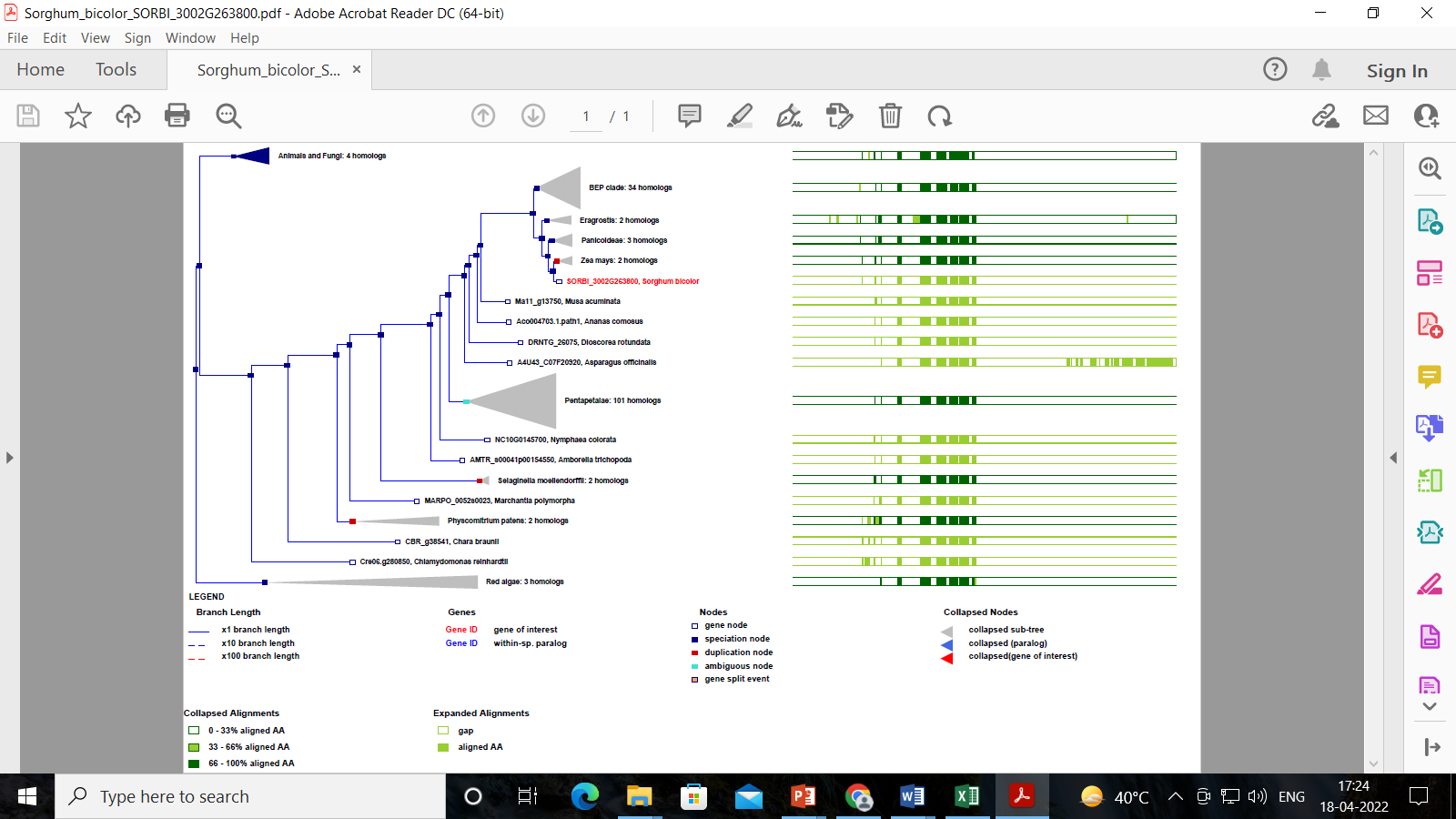
**

**Supplementary figure 1.** Ensembl plants gene tree pipeline showing orthologous and paralogous relationship of SbPA and SbPB gene with other taxa. Duplication nodes are shown as red squares whereas speciation nodes are in blue.


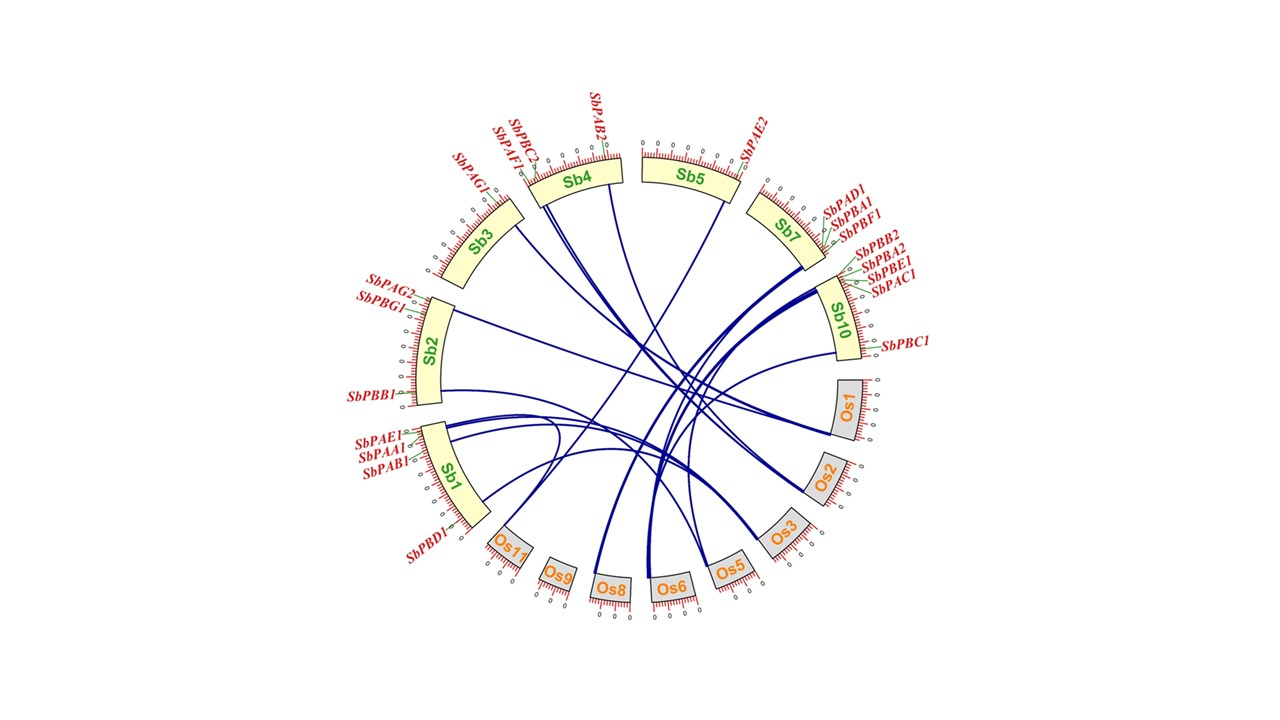


**Supplementary Figure 2.** A circular map showing synteny among PA and PB genes of *Sorghum bicolor* and Rice (name of chromosomes given outside the circle, name of genes given outside and inside the circle).


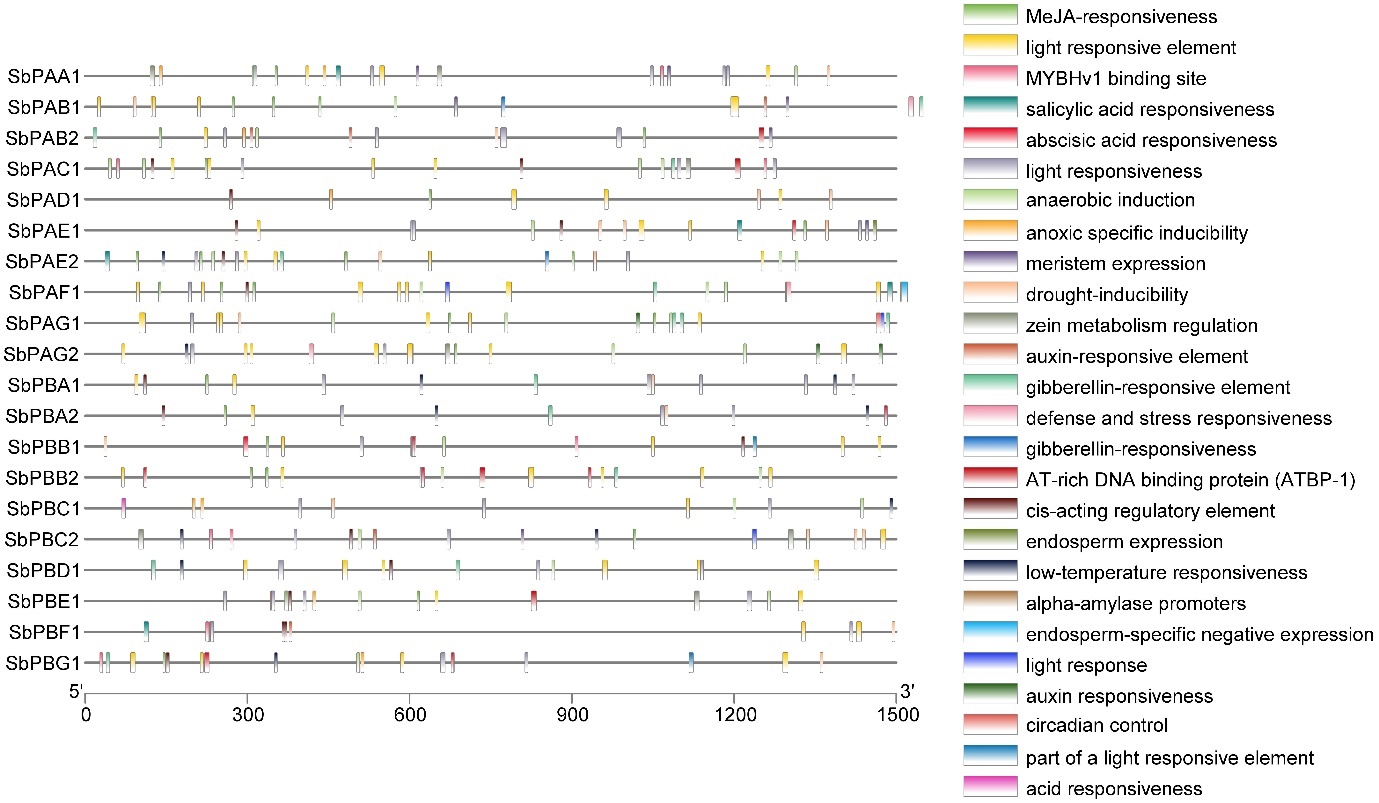


**Supplementary figure 3.** Promoter structure analysis and functional annotation of cis-regulatory elements in *S. bicolor.*


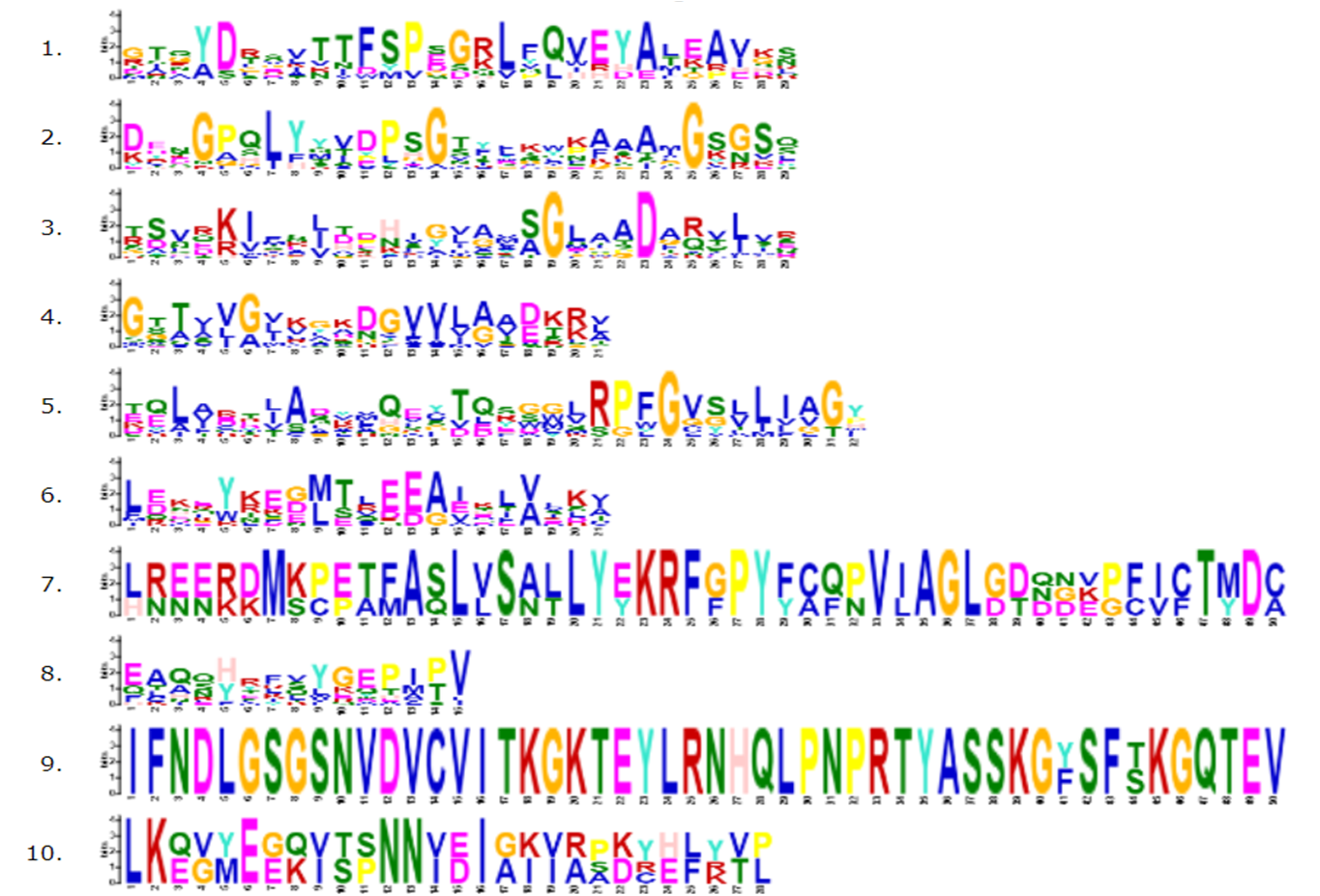


**Supplementary figure-4:** Logo of each of the 10 motifs and associated amino acids identified in sequences of *SbPA/SbPB* proteins using MEME program (relative heights of letters indicate their frequencies and level of conservation). The X-axis represents the length of motif and Y-axis represents the sequence conservation per site (bit score) of each letter.


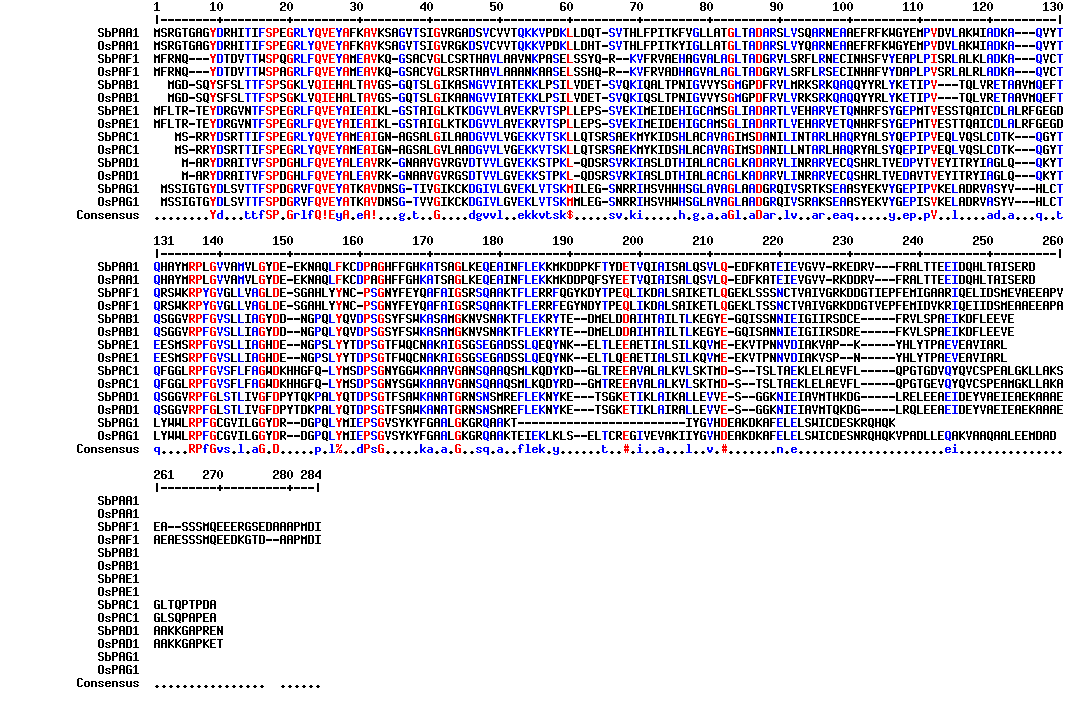


(a)


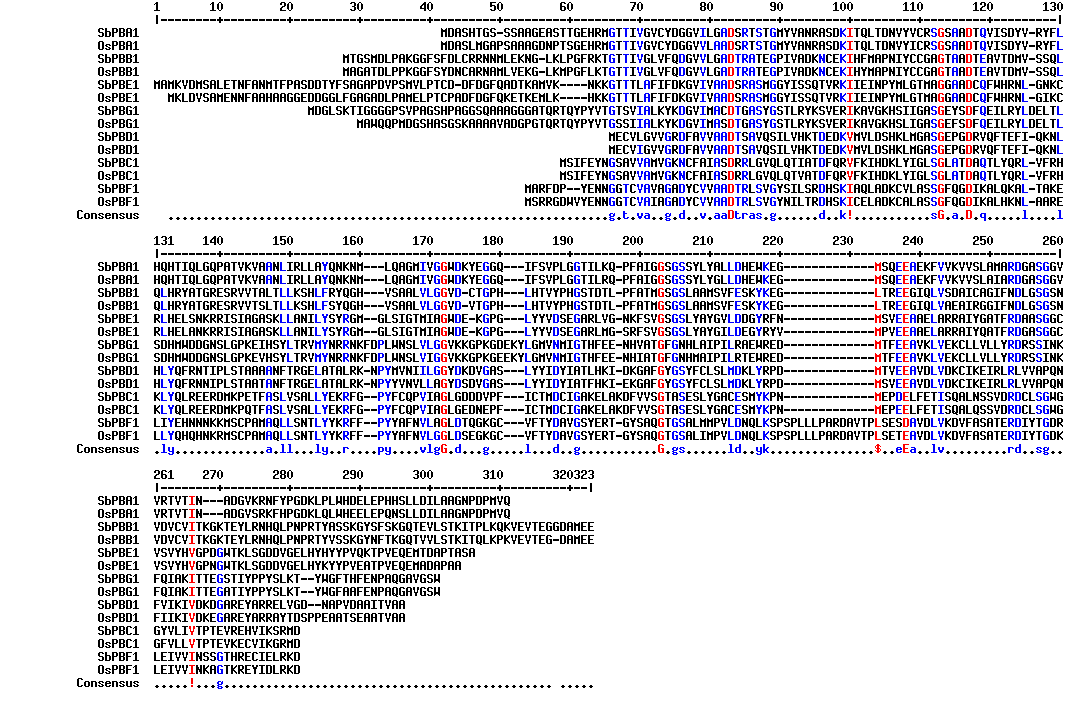


(b)

**Supplementary Figure 5 a & b.** Amino acid sequence alignments of the seven α (a) and seven β (b) subunits each of the of 20S proteasome of *Sorghum bicolor*, and rice.


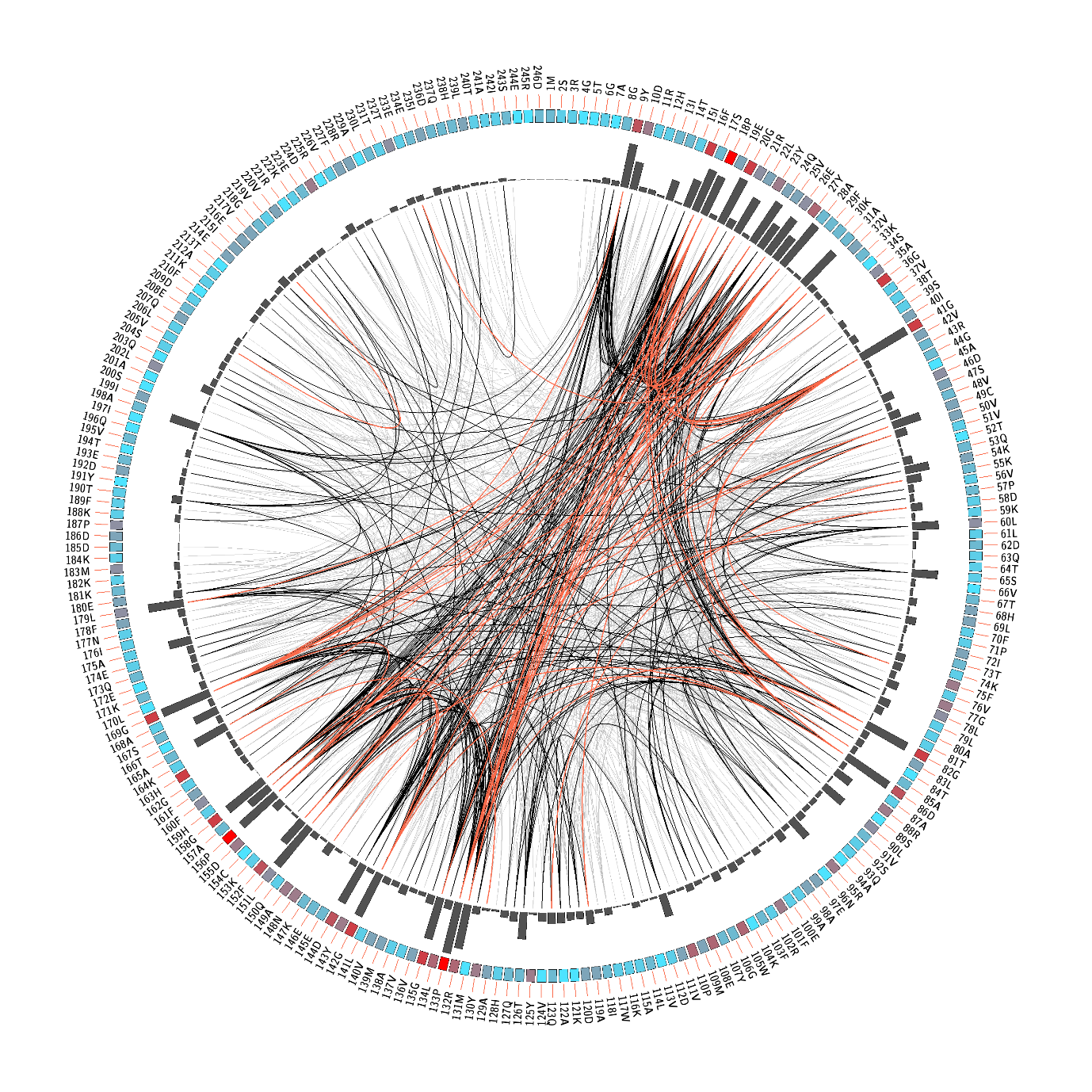


**Supplementary Figure 6.** Conserved and coevolved amino acid residues in α-subunit proteins in *Sorghum bicolor*. Mutual information network, showing conserved and coevolving residues. The labels on the exterior of second circle indicate amino acid positions of α-subunit proteins of *Sorghum bicolor.* The coloured square boxes in the second circle indicate conservation intensity (red indicates high conservation, while less conserved positions are indicated in blue). The third circle show the proximity mutual information (PMI). Curved lines in the centre connect pairs of significant MI values (> 6.5), with red lines indicating the highest MI scores (top 5%), black lines indicating midrange scores (between 70 and 95%), and grey lines indicating the lowest scores (the remaining 70%) as defined by MISTIC.


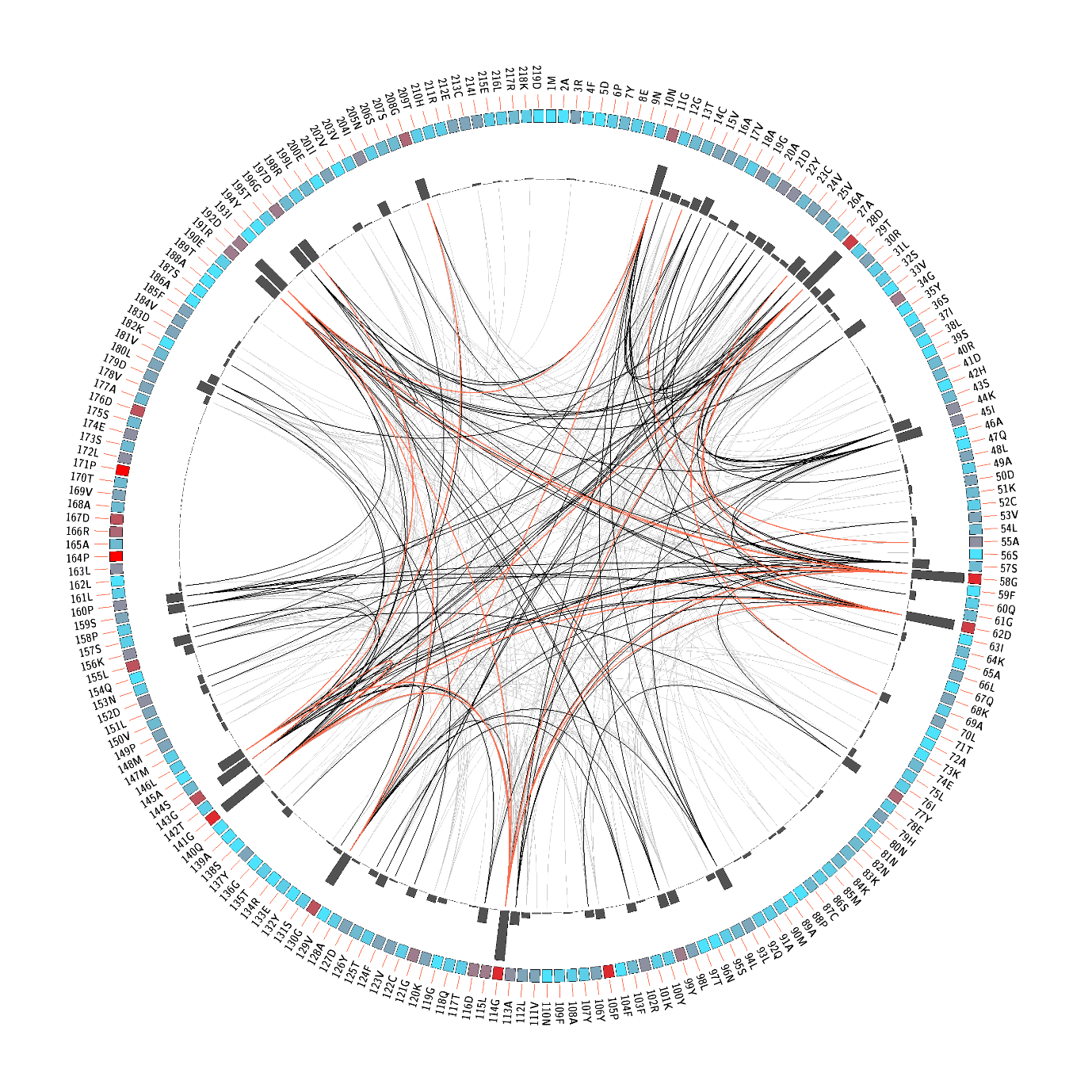


**Supplementary Figure 7.** Conserved and coevolved amino acid residues in β-subunit proteins in *Sorghum bicolor*. Mutual information network, showing conserved and coevolving residues. The labels on the exterior of second circle indicate amino acid positions of β-subunit proteins of *Sorghum bicolor.* The coloured square boxes in the second circle indicate conservation intensity (red indicates high conservation, while less conserved positions are indicated in blue). The third circle show the proximity mutual information (PMI). Curved lines in the centre connect pairs of significant MI values (> 6.5), with red lines indicating the highest MI scores (top 5%), black lines indicating midrange scores (between 70 and 95%), and grey lines indicating the lowest scores (the remaining 70%) as defined by MISTIC.


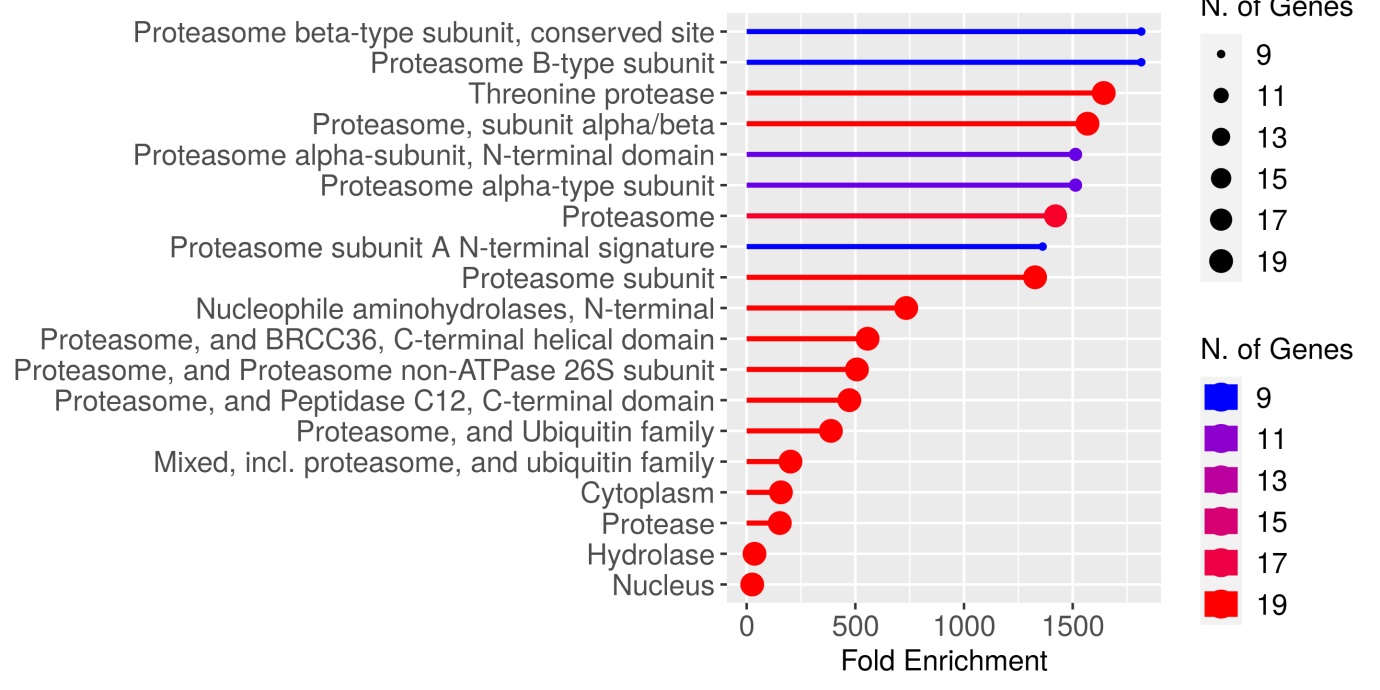


**Supplementary Figure 8.** Cellular location and biological/molecular functions of SbPA/SbPB proteins in *Sorghum bicolor*.


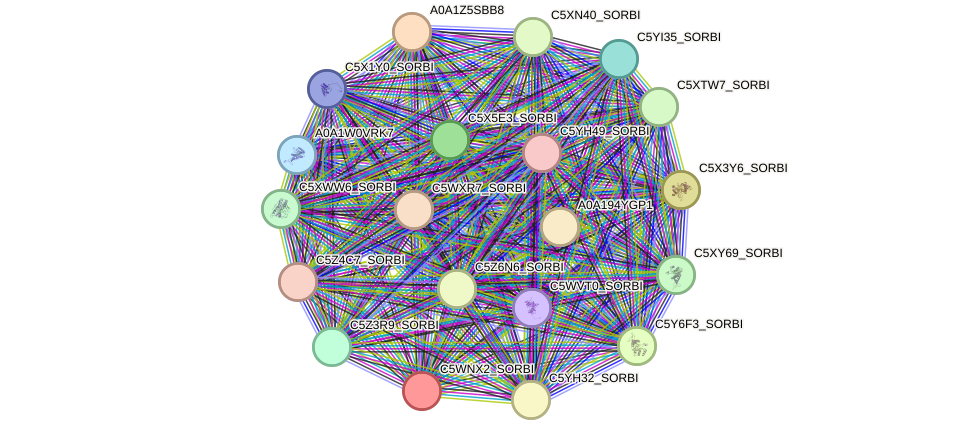


**Supplementary figure 9.** Diagram depicting protein-protein interaction network of proteasomal genes in *Sorghum bicolor* obtained through STRING database.


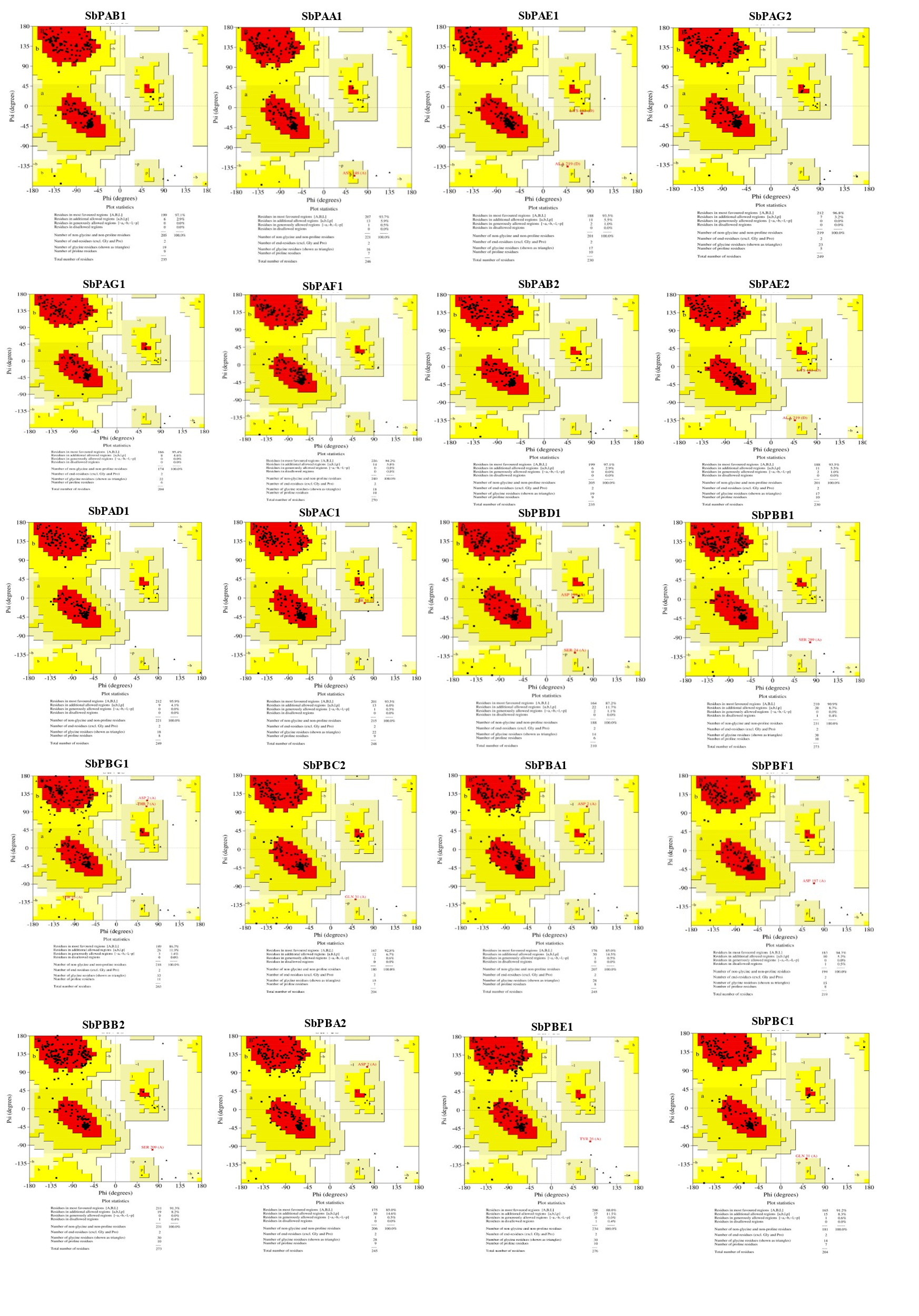


**Supplementary Figure 10.** Ramachandran plots of proteins prepared for dihedral analysis through PROCHECK

**
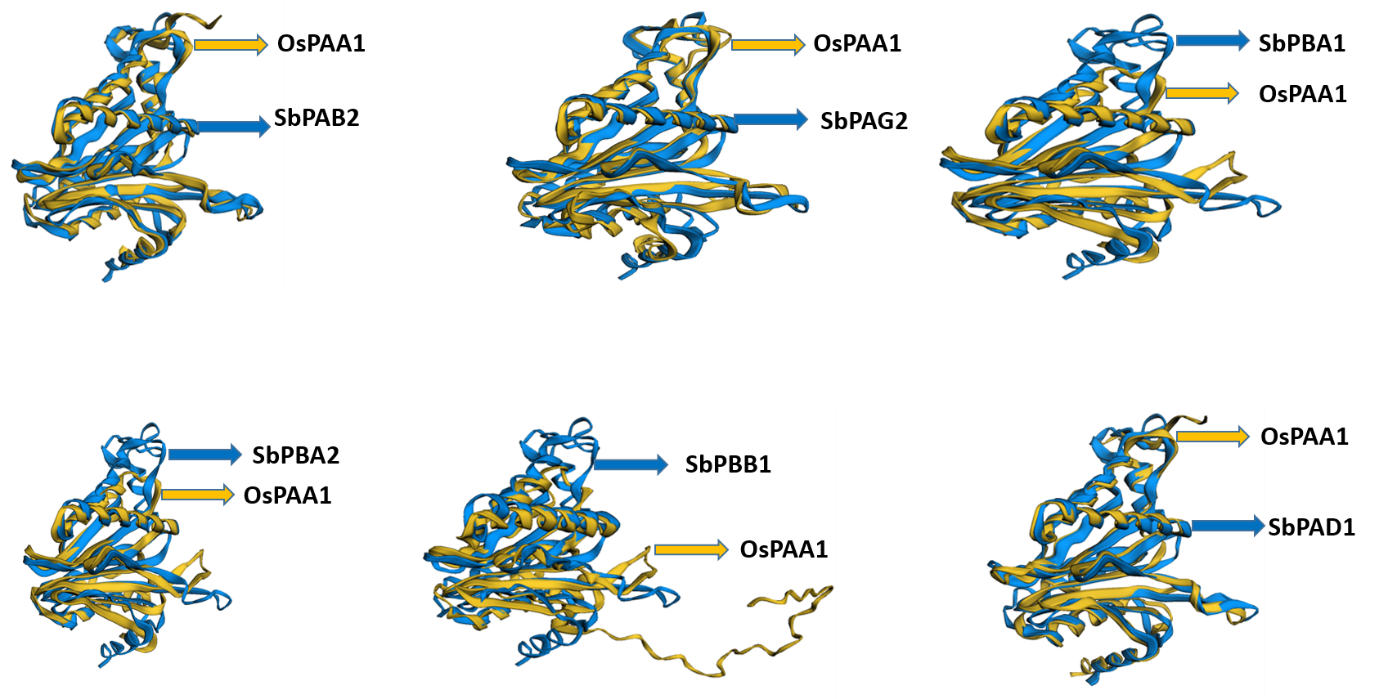
**

**Supplementary Figure 11.** Representative figure showing superimposed structure of the predicted 3D structure of SbPAB2, SbPAG2, SbPBA1, SbPBA2 and SbPBB1 proteins over 3D structure of *Oryza* PAA (OsPAA1) proteins.
